# Appreciating visual arts may not foster medical diagnosis skills

**DOI:** 10.12688/f1000research.129219.1

**Published:** 2023-01-19

**Authors:** Koji Matsumoto

**Affiliations:** 1 Faculty of Economics, Nagoya Gakuin University, 1-25, Atsuta-nishi-machi, Atsuta-ku, Nagoya, Aichi, 456-8612, Japan

**Keywords:** visual arts appreciation, diagnostic skills, observation skills, expertization, perceptual learning, mental images, pattern recognition, intuition

## Abstract

Background: This article examined intervention studies that used appreciation of visual arts to foster observation skills and discussed their effectiveness in making accurate diagnoses in terms of expertization.

Methods: In order to collect journal articles and academic books (written in English) on empirical intervention studies that examined the use of visual arts for cultivating observation skills in health professionals’ education and training, the author first targeted articles that had been included in previous systematic reviews. In addition, they conducted a manual search. From this body of literature, the author selected studies that objectively measured observation skills only through the appreciation of visual art. They collected and read around 300 articles and selected 12 studies after applying the inclusion and exclusion criteria.

Results: This article revealed no concrete evidence on whether appreciating visual art contributes toward an accurate diagnosis. Extant studies determined that such appreciation facilitates the observation of more visual features and a detailed view over time. However, they did not confirm the positive effects of appreciating visual arts on an accurate diagnosis. This article also confirmed that such appreciation does not reduce misdiagnoses or develop tolerance toward ambiguity that prevents premature closure. Moreover, the transfer of observation skills from one context to another is unlikely to be as successful as the intervention studies had intended.

Conclusions: For fostering diagnostic skills, providing students with many instances of medical cases and appropriate knowledge to evoke implicit learning for extracting subtle differences in the cases, should be prioritized over visual art appreciation. On the other hand, such appreciation may foster verbalization skills and understanding or extraction of the patient’s background and context. These competencies may cultivate teamwork and perspective-taking, indirectly leading to an accurate diagnosis.

## Introduction

Observation is crucial in various areas or specialties of medicine ^1^^–^^4^ and it is necessary for medical advancement. ^5^ Though medical education has not included the cultivation of observation skills until recently, ^2^^,^^6^ intervention studies on such training have been growing rapidly, sometimes in collaboration with art museums. ^7^ These intervention studies usually use visual arts, such as painting, based on the assumption that there are similarities between observing visual arts and making observations in medical examination or potential applications of appreciating visual arts in making medical observations. ^6^^,^^8^^–^^11^ For example, Jacques *et al.* ^12^ ^, p.9^ asserted that “the arts foster many of the same skills required of physicians, including risk-taking, problem-solving, careful and attentive looking, interpretation and analysis, developing empathy for others, and seeing an issue from multiple perspectives.”

Gelgoot *et al.* ^13^ and Elhammoumi and Kellam ^14^ reviewed several studies on developing observation skills in medical or nursing education; however, they only listed the main findings without discussing them. Additionally, Perry *et al.,* ^15^ Mukunda *et al.,* ^16^ and Alkhaifi *et al.* ^17^ concluded that such studies offer reasonable evidence in favor of using visual arts to improve observation skills. Dalia *et al.* ^18^ reviewed the incorporation of visual arts in medical school and residency and identified the following four elements that should be included in the curriculum: pattern recognition, deep seeing, facial expression, and pertinent negatives. Though Turton *et al.* ^19^ reviewed the intervention studies that used various forms of art for training in palliative care, their conclusions cannot be validated because they did not report all the literature that was used. Ike and Howell’s ^20^ review is the only known study on intervention studies that used visual arts to develop observation skills among medical professionals. However, they only discussed the validity of the psychometric scales or quantitative observation metrics used in these studies.

Thus, although one of the primary goals of developing observational skills through visual arts appreciation should be to develop diagnostic skills, it remains unclear whether appreciating visual arts is effective for improving the accuracy of diagnoses. The present article provides an overview of intervention studies that used the appreciation of visual arts for fostering observation skills and discusses their effectiveness in making accurate diagnoses in terms of the expertization of medical professions. Medical experts can diagnose more accurately through the expertization. ^21^^–^^23^ Hence, an analysis of expertization studies should provide rich insights.

The following sections of my paper are structured as follows. The Methods section explains the process of selecting the target articles. The Results section provides an overview of the intervention studies that considered the appreciation of visual arts for fostering observation skills. The Discussions section presents the findings of the target articles in terms of the expertization of medical professions. Finally we present the study’s main conclusions, limitations, and implications for future research and practice.

## Methods

### Search strategy and selection criteria

This study referred to the search results of the aforementioned review studies ^13^^–^^20^ to search journal articles and academic books (written in English) on empirical intervention studies that used visual arts for cultivating observation skills in health professionals’ education and training. Manual searches for additional articles were also conducted because the search results were inconsistent; moreover, it was difficult to collect information from databases by using typical search words such as “visual arts,” “observation skills,” and “medical education.”

Detailed inclusion and exclusion criteria to assess the results of the interventions in which only appreciating visual arts affected observation skills were set, comprising those that used only visual arts to foster observation skills. Therefore, studies that included other activities were excluded, except for pre- and post-tests, such as drawing or creating visual arts and observing medical images. Additionally, studies on “graphic medicine” ^24^ were also excluded, because these studies used comics that included written dialogues.

Moreover, studies that objectively measured observation skills were included, such as using psychometric scales, quantitative metrics, or grading participants’ statements by predetermined criteria. Thus, studies that subjectively measured observation skills, irrespective of the method used (e.g., participants’ subjective evaluation of their skills were excluded, their free comments on the interventions, and scales of their impressions of the interventions). For example, studies that extracted key concepts from the participants’ comments, such as thematic analysis ^25^ were excluded because they are not suitable for determining the learning effects for the entire concerned population since the concepts are identified from the comments of only a few participants.

### Data collection

The full texts of the related articles identified by the search results of the aforementioned review studies ^13^^–^^20^ were collected. Through manual search, the titles of articles that seemed to use visual arts appreciation in health professionals’ education were selected by checking reference lists and the articles listed in PubMed’s sidebar or Google’s search result of related articles. Then, their abstracts were reviewed via websites, such as PubMed and Google. If the abstract met the inclusion criteria, the full-text article was collected. Additionally, if the abstract could not be obtained or was insufficient to determine whether the article met the inclusion criteria, the full-text article was collected. Therefore, if it could be clearly determined from the abstracts that the inclusion criteria were not met, full-text articles were not collected. This process was repeated until no more related articles could be found. I conducted this search from January to June 2022.

### Data analysis

After a review of the full texts that were collected, studies that met the inclusion criteria were selected. An exhaustive summary of each study was collated. The summary described the features, research design, and key results of the study (see Table 1).

**Table 1.** The features, research design, and key results of the selected intervention studies.

Reference No.	Authors	Participants ( *N* is the number of samples analyzed)	Time/Duration/Frequency	Types of visual arts used	Number of visual arts used	Main activities/Protocol of the intervention	Including pair/group discussion	At an art museum	Instruction to look at the whole image in detail	Instruction to separate interpretation from description	Teaching about visual elements	Other distinctive instructions	Control experiment	Pre- or/and post-test	Timing of the pre-/post-test	Measurement of observation skills	Scoring criteria	The measurements rating the number of features/words answered/used	The measurements assessing accurate diagnoses	Key results
26	Agarwal *et al.* (2020)	1st year students in a medical school ( *N*=101)	3h×2 sessions over 2 weeks	All narrative art with no abstract objects	6	VTS	✓	✓	✓				Yes: recruited separately (intervention or doing nothing)	Yes (different but similar-in-theme images in each test)	Pre: prior to the sessions, post: the day after the last session	Electrocardiograms and chest radiographs to describe observations of differences between normal and abnormal, and a patient image to describe any observations	Frequency of clinical observations, diagnostic comments, general patient observations, and “self-deprecating” remarks/Time spent	✓ (clinical observations, diagnostic comments, general patient observations)		Some post-test scores (clinical observations and general patient observations) of the intervention group increased. Intervention group spent a statistically significantly greater time than the control group.
27	Dolev *et al.* (2001) ^a^	1st grade students in a medical school ( *N*=90, but it is unclear whether the data were obtained from all)	Not reported	Representational paintings that the students were not familiar with	Not reported	Individual and group descriptions of visual evidence in paintings	✓		✓	✓			Yes: randomized voluntary applications (intervention/clinical tutorial sessions of history taking and physical examination skills/anatomy lecture that featured abdominal radiographic images)	Yes (different photos in each test)	Not reported	Patient photos were presented to write descriptions of what they observed, not a diagnosis or any pathophysiologic process (the number of artwork and images used was not reported)	A key that assigned 1 point for each of the nine or 10 visual diagnostic features present in each photograph	✓		The intervention group’s post-test score improved by 56% than the pre-test and it was 10-12% higher than the other groups.
28	Garino (2008)	Physician associate students of a school in 2008 and 2009 ( *N*=29, 36, respectively), before dermatology and physical exam training	2 h×1 session (a mandated element of the curriculum)	Not reported	Average of 4 works for each student	Participants were asked to describe the paintings individually and in groups	✓	✓		✓			No	Yes (different photos in each test)	Not reported	Three medical photos depicting physical abnormality were shown and participants were asked to describe what they observed, ranged from patient portraits to studies of an isolated anatomical structure	A key that assigned 1 point for correctly identified item out of all 10 items in each photo. Items ranged from the very general to the very specific (lesion detail)	✓		Significantly improved in 2008 (14.07 to 15.69) but not in 2009 (14.00 to 14.63)
29	Goodman & Kelleher (2017)	1st year radiology residents in a school ( *N*=15)	1 session (length of the session was not reported)	Paintings from the gallery’s collection	Not reported	Describing the painting, then interpreting it	Not reported	✓	✓	✓			No	Yes (different images in each test)	Pre: the first day of their residency (timing of the session was not reported), post: immediately after the session	Fifteen radiographs were shown to identify the location of abnormality, but the participants were asked to not present a diagnosis	Whether the abnormality was identified or not		✓	Improved (2.3 to 6.3)
30	Griffin *et al.* (2017)	Higher specialty trainees (specialist registrars) of dermatology ( *N*=4, with 3 participants in the control group)	2 h×5 sessions with additional self and group reflection (duration of the sessions was not reported)	Abstract, landscape, still life, portraiture, and figurative artwork	Not reported	Analysis of selected artwork, group discussion, short lectures, and follow-up resources	✓	✓			✓	Encouraged to challenge their own perceptions, consider the artist’s viewpoint, and develop their own descriptive style	Yes: (probably) voluntary (intervention or control, but details of the control were not reported)	No (post-test only)	Not reported	Artwork and clinical images to identify key visual features and comment on texture, lines and contour, color (or shading), and contrast (the number of artwork and images used was not reported)	Frequency of correct answers pre-set by dermatologists (clinical images) and an arts educator (artwork)	✓ (probably)		No significant difference in clinical images (Controls: 12.67; Participants: 13.25) and artwork (Controls: 43.00, Participants 60.75)
31	Grossman *et al.* (2014)	Family nurse practitioner program students in a school ( *N*=19, but it is unclear whether the data were obtained from all)	2 sessions held a month apart	Paintings with rich details and multiple possible interpretations. The students were unfamiliar with all the paintings	Not reported	Group activities to describe the paintings and discuss the interpretations based on the descriptions	✓	✓	✓	✓			No	Yes (different images of the same futures in each test)	Beginning and end of each session	Two photographs of dermatological lesions were shown and participants were asked to describe and interpret them	Lists of observations and interpretations	? (because the scoring procedure was not reported)		Post-test scores of both observation and interpretation for Session 1 were significantly higher than pre-test. Post-test scores for Session 2 were higher (but not significantly) than those of Session 1.
32	Gurwin *et al.* (2018)	1st year students in a medical school ( *N*=36, but it is unclear whether the data were obtained from all)	1.5 h×6 sessions (1 session per week)	Not reported	Not reported	Artful Thinking approach	Not reported	✓	✓	✓	✓		Yes: randomized (intervention or control that did not receive any training but received free membership to the museum)	Yes (it is not known whether the same or different images were used in each test)	Pre: before randomization, post: end of the study period	Art images and two kinds of medical images were shown for observation (the number of images used was not reported)	Art image: frequency of using terms related to critical thinking skill. Medical images: frequency of observations and general quality of the description	✓ (medical images)		The intervention group in the post-test outperformed the control group and their pre-test scores for all the images.
33	Huang *et al.* (2016)	Dermatology house officers in a training program ( *N*=27, but it is unclear whether the data were obtained from all)	Introductory 3 h + 1.5 h×3 sessions over 2 months	Not reported	Not reported	VTS + brief homework based on the session’s theme	✓	✓	✓		✓	Complementary themes, including the exploration of the assumptions and approaching ambiguity	No	Yes (different images in each test)	Not reported	Three clinical images and two art images were presented for observation	Frequency of unique, accurate observations per image	✓		Significantly increased (all images: 28.7 to 33.1)
34	Jasani & Saks (2013)	3rd year students (probably) in a medical school ( *N*=70)	3 h×1 session	Both representational and non-representational art	8	4-step method customized VTS (observation, interpretation, reflection, communication)	✓		✓	✓			No	Yes (it is not known whether the same or different photos were used in each test)	Not reported	Two patient photos were shown and participants were asked to list unique observations regarding the first and write a free text description about the second	First: frequency of observations. Second: frequency of use of subjective terminology, scope of interpretations, speculative thinking, and use of visual analogies	✓ (first photo)		First: no significant difference. Second: decrease in subjective descriptions, increase in scope of interpretations and use of speculative thinking, and visual analogies
35	Klugman *et al.* (2011)	Students (1st to 3rd year) from a medical school and a nursing school (undergraduates and graduates) ( *N*=23)	1.5 h×3 sessions (one session per week)	Paintings including less representational works	9	VTS	✓	✓	✓			In the 3rd week, participants were asked to focus on interpreting the emotional quality of works	No	Yes (randomly assigned to receive one of the two versions as the pre-test and the other as the post-test)	Pre: 2 weeks before the initial session, post: 1 week after the end of the program	Three images of artworks and three images of patients in dermatology were presented and participants were asked to write what they observed	Number of words used for the description/Frequency of single factual declaration	✓		Increased in both criteria of both images
36	Pellico *et al.* (2009)	An accelerated master’s program of nursing ( *N*=66)	1.5h×1 session	Paintings including allegorical pieces	Not reported	Group activities to describe the painting and discuss the interpretations based on the descriptions	✓	✓	✓	✓			Yes: (probably) randomized (the control: teaching clinical learning strategies in the traditional classroom)	No (post-test only)	Not reported	Six patient photos were shown and participants were asked to write what they observed and their interpretations of the clinical issue represented in the photos	Written word count, frequency of plausible objective clinical findings, frequency of alternative diagnosis offered	✓		The intervention group mostly outperformed in every criteria
37	Poirier *et al.* (2020)	Students from an undergraduate honors level course on health, not all health care majors ( *N*=17)	10 to 15 minutes were spent at the beginning of the class at least once per week	Various images of art mediums and styles	25	VTS			✓				No	Yes (same photos in both the tests)	Pre: beginning of the course, post: end of the course	Three paintings were shown and participants were asked to describe them using VTS questions	Frequency of descriptions of name or identifying something, frequency of descriptions of a single factual declaration	✓		No significant improvement (26.82 to 29.00/17.59 to 19.65)

^a^ This review only covers the intervention that took place between 1998 and 1999.

On the basis of the summary, the features and tendencies were synthesized into the interventions’ goals and methods, research design, and findings of the studies. Then, the features and tendencies were examined in relation to the findings of the studies on expertization of medical diagnostic skills and visual perception, according to review articles on related themes as well as the articles discussed in those reviews. Through the examination, the aforementioned review studies ^13^^–^^20^ and relevant articles that were collected but excluded in this study were also referenced.

## Results

A total of around 300 articles were reviewed in detail. After applying the aforementioned inclusion and exclusion criteria, the literature search yielded 12 studies, ^26^^–^^37^ as listed in Table 1. Thereafter, 3 of these 12 studies ^28^^,^^31^^,^^37^ were found by manual search and missed by search results of the aforementioned review studies. ^13^^–^^20^ Five studies ^38^^–^^42^ were excluded from the analysis because they included activities other than visual arts appreciation.

The intervention studies were conducted for under- or post-graduate students in various disciplines, such as radiology, dermatology, and nursing. They were implemented in a specific school, hospital, grade level, program, or class. Sample sizes in these studies were relatively small. The frequency of sessions in each study was diverse, ranging from a single session lasting only a few hours to multiple sessions over two months. The intervention studies were often implemented in art museums.

Most studies used paintings as visual art and asked the participants to describe and discuss the paintings’ details and their interpretations in pairs or groups. Some of the interventions utilized Visual Thinking Strategies (VTS), embodying the following three core questions: “What’s going on in this picture?,” “What makes you say that?” and “What else can we find?” These questions help participants to observe the entire painting carefully and make inferences based on the visual evidence. ^43^ Other interventions focused on separating the description of visual evidence from the interpretation or using the concepts of visual elements, such as color, light, and texture.

Most studies used control experiments or pre- and post-tests to examine participants’ descriptions of visual features in paintings or medical images. They revealed that the intervention enabled participants to describe what they saw in more detail and use more words. Some studies, however, did not show this effect, possibly due to few participants, disagreement between scorers, or a ceiling effect on the measurements. Concerning the qualitative aspects of participants’ descriptions, Jasani and Saks ^34^ observed an increase in descriptions through the use of visual analogies and scope of interpretations that involved the patient’s surroundings, perspective, or emotional state. They also observed an increase in the number of words used to express speculative thinking, which indicated the possibility of multiple interpretations. Furthermore, Agarwal *et al.* ^26^ reported that the intervention group spent a statistically significant and greater amount of time observing and describing than the control group.

Of these studies, only Goodman and Kelleher ^29^ directly measured diagnostic accuracy. They used the identification of abnormalities in the radiographs and reported that their intervention was successful. However, the correct identification rate was less than half, even though the abnormalities were conspicuous and of a low level of difficulty.

## Discussions

### Longer periods of careful and systematic observations

Most interventions instructed participants to carefully observe the entire target. As stated by Agarwal *et al.,* ^26^ taking time to observe is considered a sign of careful observation. However, observing for a longer period is not necessary for an accurate diagnosis. Experts take less time than novices to diagnose accurately. In contrast, they are more likely to misdiagnose if they take longer. ^44^^–^^46^

Moreover, systematic viewing intervention, such as detecting radiograph abnormalities by viewing the images in a given order, or full-coverage viewing intervention, such as mentally dividing each image into nine imaginary segments (3×3) and inspecting each segment separately, did not outperform non-systematic viewing intervention, wherein participants were urged to inspect whatever attracted their attention. ^47^ Systematic and full-coverage viewing took a longer (but not significant) time than non-systematic viewing. Van Geel *et al.* ^48^ also reported similar results.

Many studies ^44^^,^^49^^–^^51^ and reviews ^52^^–^^58^ on medical image perception have shown that experts’ diagnosis is generally characterized by holistic and quick recognition. This is due to pattern recognition ^59^^–^^61^ or intuition composed of scripts, schemas, or mental models/images/representations (hereinafter collectively referred to as mental images). ^62^ Experts compare medical images or patients with mental images—which are associated with domain-specific knowledge—of normal and abnormal cases at a single glance. ^23^^,^^63^^–^^65^ A study using fMRI ^66^ revealed that when radiologists looked at radiographs, the brain regions related to visual attention and the encoding, storing, and retrieval of visual memory were activated.

Not just medical images, humans generally recognize things by associating them with mental images formed through our daily visual experiences. ^67^ For example, humans can recognize a scene’s gist and gaze into areas of interest through their knowledge of the world. ^68^^–^^70^ This top-down control of gaze by using mental images is common for humans and is not limited to those who diagnose medical images.

The experts’ intuition is cultivated through the accumulation of domain-specific knowledge and experiences. The number of mammograms observed is proportional to the accuracy of the diagnosis. ^71^ Thus, it is not conducive if the intervention studies do not consider the participants’ pre-existing knowledge in measuring observation skills.

### Cultivating tolerance for ambiguity

For encouraging persistent and detailed observation, some interventions ^33^^,^^35^^,^^72^ (included in this article and others) are intended to cultivate tolerance for ambiguity. An increase in speculative thinking—which was observed by Jasani and Saks ^34^—implies such cultivation.

However, the intervention studies ^35^^,^^72^ showed that using visual arts contributes very little or does not contribute toward building tolerance for ambiguity.

### Discovering more visual features or signs

The intervention studies enabled the participants to describe the visual features in the paintings and medical images in greater detail. However, the observation and description of more visual features or signs in medical images or patient photographs do not lead to accurate diagnoses for novices and experts. Novices, such as medical school students, generate hypotheses based on constant information input and are unable to select appropriate hypotheses. ^73^ An experiment that presented electrocardiograms to non-medical laypersons revealed that participants usually misdiagnosed the problem because they were unable to overlook irrelevant features. ^74^

Not just in the context of medical images, experts generally diagnose and make decisions based on mental images. ^75^ They can extract more useful information about the given situation, immediately generate appropriate hypotheses, eliminate irrelevant hypotheses, and test the hypotheses. ^62^^,^^63^^,^^76^ Thus, expert general practitioners can make a correct diagnosis without discovering all the features of a disease. ^60^

### Observing carefully for reducing misdiagnoses

Goodman and Kelleher ^29^ asserted that reducing misdiagnoses is one of the benefits of visual training, such as appreciating visual arts. Though they did provide an explicit reason for why visual training would reduce misdiagnoses, it can be asserted that misdiagnoses arise from careless observation, and appreciating visual arts can teach one to look carefully.

However, the causes are complex ^77^ and may not always be due to mere carelessness. In general, medical errors can be classified into search errors caused by not looking at the abnormality, recognition errors caused by not identifying an abnormality, and decision errors that occur in the process of clinical reasoning. ^78^

In the context of novices, Brunyé *et al.*’s ^78^ review showed that search errors generally occur when novices overlook definitive features with subtle visual features and look repeatedly at areas that are not relevant for a diagnosis. Misdiagnoses is also called when signs of extremely low prevalence are overlooked by novices (and experts). Novices are also likely to make recognition errors due to insufficient mental images and knowledge. ^49^^,^^65^ This implies that novices’ errors are caused by the lack of top-down control of gaze due to insufficient mental images.

On the other hand, experts can diagnose quickly and accurately by using mental images. Mental images are compromises between fitting the data and minimizing the complexity of the model, ^67^ which leads experts’ perceptions toward certain classes of stimuli in the domain. ^23^ Therefore, many expert radiologists overlook the gorilla inserted into chest CTs. ^79^ This demonstrates that experts’ errors, such as premature closure, may be a byproduct of expertization. ^23^^,^^80^

Furthermore, human and environmental factors that induce decision errors cannot be ignored. ^81^ Therefore, cultivation of novices’ mental images, organizational/systematic support or use of cognitive tools/strategies ^82^ may be more effective than appreciating visual arts to reduce medical errors.

### Transfer of observation skills to identifying different objects

The intervention studies intended to transfer observation skills for the visual arts to apply to medical examination, based on the assumption that there are similarities between these fields. However, the transfer occurs only to a very limited extent. Experimental studies showed that in terms of radiology, expertise was positively transferred to the task of finding small low-contrast dots in phantom X-ray images ^83^ but not to the task of finding hidden targets in pictorial scenes. ^84^ The same can be said for visual recollection memory. Expert cytologists and radiologists were not better at recognizing scenes or isolated objects than non-medical participants. ^85^

The limitations of the transfer depend on the domain-specificity of mental images. Whether looking at medical images, patients, landscapes, or paintings, each requires unique domain-specific knowledge and experiences to form mental images.

### Is there a more effective way?

I suspect that the effectiveness of Goodman and Kelleher’s ^29^ intervention is unsatisfactory. Their task was too easy, as making a diagnosis is more difficult than identifying abnormalities. ^86^ There may be more efficient or effective ways of fostering diagnosing skills than appreciating visual arts. For example, learning modules, based on theories of perceptual learning, such as Perceptual and Adaptive Learning Modules (PALM), have been developed to foster pattern recognition of medical images. ^61^^,^^87^ All the relevant studies have reported excellent results of diagnostic accuracy for medical school students and/or residents ^22^^,^^88^^–^^94^ and non-medical participants, ^95^^–^^98^ however, it is possible such excellent results across the board could be due to publication bias (see also the review by Guégan *et al.* ^99^). Although it is difficult to directly compare the studies due to different testing methods, these modules seem superior to Goodman and Kelleher’s intervention. ^29^ It is also notable that the outcome measures used by these modules included fluency, or the speed of recognition. This is consistent with research findings on expertization.

Likewise, teaching a specific eye movement search pattern that corresponds with the characteristics of the area shown in the radiographs ^100^ or demonstrating an expert model that searches visually and interprets symptoms (eye-movement modeling examples) ^101^ could also be effective.

### Fostering verbalizing skills for diagnoses

It would be more accurate to say that the interventions that use the appreciation of visual arts foster verbalizing skills. It also may be useful to teach concepts of visual elements, such as color, light, and texture in order to foster such skills. Verbalizing accurately and lucidly is important for various reasons in medical practice. ^102^

Group discussions can also act as an opportunity to practice and reflect on one’s verbalization skills. This can contribute toward the development of communication skills that are required for teamwork. Klugman *et al.* ^35^ intended to develop communication skills and claimed that their intervention achieved the same objective. However, they did not present any empirical data to support their claim.

The use of visual arts may also be useful for group discussions across specialties. For example, Katz and Khoshbin ^103^ reported that their curriculum included visual arts to facilitate multidisciplinary team building. One possible reason for this was that the visual arts free the participants from the hierarchy of medical personnel and ensure their equal participation in discussions. It has been reported that even children who do not perform well academically or usually speak, actively participated in the VTS sessions. ^43^

### Considering patients’ contexts and perspective-taking

Thus far, I have shown that appreciating visual arts may not have a direct effect on making an accurate diagnosis. Meanwhile, Jasani and Saks ^34^ observed an increase in descriptions of the scope of interpretation. Understanding the patient’s background and context—which are invisible—may be necessary for patient interviews. This corresponds with Shapiro *et al.*’s supposition ^104^ that teaching through cases would be effective in fostering pattern recognition, while teaching through paintings would promote interpretation skills based on a broader context, including a patient’s subjective perception and understanding of diseases.

Perspective-taking as cognitive empathy, such as knowing and postulating how another is thinking and feeling, ^105^ is also essential for patient interviews. Appreciating visual arts and group discussions about observations and interpretations of them may foster such perspective-taking. Although there are several reports on such practice, ^106^^–^^111^ none presented empirical evidence. On the other hand, Gurwin *et al.* ^32^ used the Reading the Mind in the Eyes Test, ^112^ which assesses the ability to recognize emotions based upon photographs of actors’ eyes, and reported no significant difference, possibly due to the ceiling effect. It would be beneficial to consider using other measures, such as the Theory of Mind Test ^113^ and Faces Test, ^114^ as exemplified by Pino and Mazza. ^115^

Understanding patients’ facial expressions is also likely to enhance perspective-taking for medical professions. ^18^ It may be worthwhile to train such understanding because of the functional specialization for face perception in the brain. ^116^ While several studies have reported on such practices, ^117^^,^^118^ they did not provide any supporting empirical evidence.

## Conclusions

This article revealed that there is no concrete evidence on whether appreciating visual art contributes toward an accurate diagnosis. Although the appreciation of visual arts has helped facilitate the observation of more visual features and the ability to see more thoroughly by taking more time, findings of the expertization studies do not prove that these contribute toward the cultivation of skills required for making a diagnosis. Additionally, the claim that such appreciation can reduce misdiagnoses or cultivate tolerance for ambiguity that prevents premature closure has not been proven. Moreover, the transfer of observation skills to different objects is unlikely to be as successful as the intervention studies intend. Rather, it would be better to use learning modules based on perceptual learning using cases to foster pattern recognition. However, such appreciation (sometimes with group discussions) may foster verbalization skills and understanding of the patient’s background and context. This may indirectly contribute toward accurate diagnoses by facilitating teamwork or fostering perspective-taking. However, the effects of such appreciation remain ambiguous.

Alkhaifi *et al.* ^17^ asserted that visual arts can be means to address important competencies that are difficult to teach using traditional methods; however, it is important to state that visual arts are not a panacea. The reasons why visual arts appreciation is unlikely to foster diagnostic skills can be summarized as follows. First, discovering more signs of a disease *does not* equate to diagnosis. Diagnosing medical images and actual patients involves determining whether the patients or medical images fit into a particular classification of diseases or not. Experts can make a correct diagnosis without discovering all the features of a disease because they focus only on useful features for such distinctions. Gibson’s theory of perceptual learning ^119^ precisely explains expertization in such distinctions. Therefore, intervention studies have assumed that medical experts’ approaches to observation are similar to those adopted by artists; however, these approaches differ because no such distinctions are necessary for artists.

Second, such distinctions are intuitive and involve implicit perpetual learning. ^120^ It is possible even if the rationale is not verbalized, although more effective if it is verbalized. ^119^ Experimental psychology has shown that simply looking at various paintings with the names of the artists at random can enable novices to differentiate between artists. ^121^^–^^123^ Similar results were obtained in experiments that had novices diagnose psychopathological cases presented visually (through words) or aurally. ^124^ Additionally, novices have been shown to be able to detect melanoma by being shown benign and malignant cases side by side, but without being given instructions, such as the ABCD rule. ^125^ Further, instructing novices on such rules has little effect on diagnostic accuracy. ^125^^,^^126^ Protocols in which participants contrast similar cases—that learning modules such as PALM also adopt—use implicit learning to extract subtle differences in the cases. Therefore, it seems difficult to enhance such implicit learning through a closer look at a piece of visual art.

Third, mental images are domain-specific. Visual arts do not substitute domain-specific knowledge or one’ s experience with medical cases.

### Limitations

This article has several limitations. First, I excluded articles published in languages other than English and those that subjectively measured observation skills. These studies may have included information that could have contributed to this article.

Second, I found only one study that directly examined the impact of appreciating visual arts on diagnostic skills. The conclusions of this article are tentative, while the findings of the expertization studies are sufficiently reliable.

Third, I was not able to fully interpret the results of Goodman and Kelleher, ^29^ specifically, the small transfer of visual arts appreciation to the identification of abnormalities in the radiograph. Although it may be due to adapting to the test format, it is not clear by what process the transfer occurred, if at all.

### Implications for future research and practice

I offer the following suggestions for future research. First, while studies on expertization reveal qualitative differences between novices and experts, the process of expertization is not understood very well. ^74^^,^^127^ It is likely, albeit to a lesser extent, that listing more visual features and hypotheses is unnecessary for the process of expertization. Hence, it may be promising to investigate whether listing such features and hypotheses by appreciating visual arts is effective in scaffolding diagnosis skills. For example, such appreciation might accelerate learning through the learning module based on perceptual learning. Thus, longitudinal studies may be required ^13^ because the effects of such appreciation may be delayed.

Second, to measure the effectiveness of appreciating visual arts, it is recommended to use the same tests that have been used to measure the effectiveness of learning modules on perceptual learning. Diagnostic accuracy and fluency should also be examined, as asserted by Kellman *et al.* ^22^ Additionally, the influence of pre-existing knowledge should be considered.

Third, as mentioned by Perry *et al.,* ^15^ the intervention methods must also be reported adequately. It is unlikely that merely looking at visual art develops the skills required for medical diagnoses. Rather, the design of the experience, such as selection of the paintings, instruction, and facilitation and activities within peers/groups is required because the outcome of the intervention should vary based on the type and number of paintings that have been viewed and on the activities or reflections mentioned as instructions. ^128^ It is also necessary to mention the responses that were evoked by the subjects (or paintings), prompts, and questions to identify whether the outcomes were intended or derived. Moreover, I found the following deficiencies in the studies used in this article: the number of missing samples from the participants; what did the control group do; details of the content and format of the lectures that the control participants were subjected to; maximum possible values of the scales or items for considering the ceiling effect; details of the scoring criteria; how many schools/organizations did the participants belong to; how many images were used for the test; and timing of the pre- and post-test. Thus, items listed in Table 1 should be reported in future studies.

Fourth, it is necessary to investigate whether the skills and knowledge that would be fostered by appreciating visual arts enhance diagnosis skills. This article found that appreciating visual arts may enhance verbalizing skills, teamwork skills, interpretive skills, and perspective-taking. Mukunda *et al.*’s ^16^ review also found a paucity of rigorous studies on training modules that use arts to promote empathy, team building, communication skills, wellness/resilience, or cultural sensitivity.

Fifth, research is needed that directly compares the skills fostered by visual arts appreciation with other methods that attempt to foster the same skills, such as learning modules applied by perpetual learning and using other forms of arts. For example, we can use only visual arts and also performing arts for teamwork and communication skill development among health professionals. ^129^ Due to time constraints imposed by the many demands of medical education and training, it is necessary to identify a more efficient method among those available.

Finally, as Osman *et al.* ^130^ suggested, further research exploring the process of learning through visual arts appreciation is required, as extant literature only focuses on outcomes. Dominant research methods such as controlled studies and psychometric scales do not allow for such investigation ^128^^,^^131^; thus, further research should include alternative methods and outcome measurements, such as qualitative methods. ^15^^,^^132^

I also offer the following suggestions to practice fostering diagnostic skills. Given the time constraints, adopting visual art appreciation is not encouraged. Taylor ^64^ claimed that education in medical school should provide students with many instances of medical cases and appropriate knowledge to form adequate mental images. Such provision must be made in an appropriate manner, as in PALM, to evoke implicit learning in order to extract subtle differences in the cases. This should be prioritized over visual art appreciation.

Furthermore, since expert perception is a combination of observation and interpretation through intuition, separating observation and interpretation, as instructed in several intervention studies, seems to be unnatural and could be avoided. Jaarsma *et al.* ^133^ showed that in clinical reasoning, experts used less descriptive terms, like expressing colors and shapes, and used more comparative terms to interpret findings in terms of normal/abnormal or typical/atypical.

## Data Availability

All data underlying the results are available as part of the article and no additional source data are required.
