# Effects of visual art observation on technical skills in novice healthcare learners: A scoping review

**DOI:** 10.12688/f1000research.129219.3

**Published:** 2026-01-24

**Authors:** Koji Matsumoto

**Affiliations:** 1 Faculty of Economics, Nagoya Gakuin University, 1-25, Atsuta-nishi-machi, Atsuta-ku, Nagoya, Aichi, 456-8612, Japan

**Keywords:** visual arts observation, diagnostic accuracy, observation skills, verbalizing skills, perceptual learning, mental images, pattern recognition, intuition, scaffolding

## Abstract

**Background:**

Recently, health professional education uses visual art observation to promote various observation-related technical skills. This article maps the studies on such interventions, scrutinizes what they measured as observational skills, and discusses their effectiveness.

**Methods:**

Following the PRISMA Extension for Scoping Reviews, a scoping review was conducted. Publications from 2001 on were identified by searching four databases and by hand searching. The author screened each publication using the pre-designed eligibility criteria: participants were novice healthcare learners enrolled in visual art observation training; the study aimed to evaluate the effect of the intervention on technical skills related to observation; the skills were objectively measured. The author extracted relevant information from the included papers without additional inquiry into the study authors. The extracted information was illustrated in both a tabular and descriptive format.

**Results:**

3,157 publications were identified, of which 18 articles were included. Few studies had valid and reliable experiments. The relatively valid evidence is that the participants listed more elements or signs for artistic or medical images.

**Conclusions:**

Sound evidence is lacking for all the technical skills intended to be fostered. Observation skills for artistic images have not been demonstrated to transfer to technical skills. Nor do the studies show that they promoted accurate diagnoses and reduced misdiagnoses. Additionally, the evidence on verbalizing skills is not isolated from the impact of discussions and is unclear regarding its transfer to actual communication. For the others, there are not enough valid studies on technical skills. This is true for studies that directly examine promoting accurate diagnosis or reducing misdiagnosis. Moreover, there may be promising alternatives to visual art observations for cultivating such technical skills, but no comparative studies were conducted.

## Introduction

Observation is crucial for advancing healthcare in various health professions. ^1^^–^^5^ Though health professional education has not included cultivating observation skills until recently, ^2^^,^^6^ intervention studies, i.e., studies which include both training practices and corroborating its effects, on such training have grown rapidly, sometimes in collaboration with art museums. ^7^ These intervention studies usually use visual arts, such as painting, based on the assumption that there are similarities between observing visual arts and making observations in medical examination or potential applications of observing visual arts when making medical observations. ^6^^,^^8^^–^^11^

Advocates argue that it can improve the following technical skills ―in this article, “technical” means unique to a specific job role― related to observation: tolerating being unsure for long enough to sustain a meaningful analysis; ^12^ grounding inferences in evidence; ^12^ assessing medical images and patient symptoms accurately to reduce misdiagnosis; ^13^^,^^14^ metacognitive awareness; ^12^ capturing and documenting clearly what is observed ^15^ in teamwork. Also, concerning accurate diagnosis in patient interviews, the skills include: gleaning nonverbal cues to support how they care for a patient, ^15^ such as reading their facial expressions ^6^; understanding or extraction of the patient’s background and contexts; perspective-taking as cognitive empathy, such as knowing and postulating how another is thinking and feeling, ^16^ for patients. ^15^ Thus, in this article, observation skills are defined broadly.

Previous reviews on the use of visual arts in developing observation skills in medical and nursing education have been limited in their scope and analysis. For example, Gelgoot *et al.* ^17^ and Elhammoumi and Kellam ^18^ reviewed several studies on developing observation skills in medical or nursing education; however, they only listed the main findings without discussing them. Though Turton *et al.* ^19^ reviewed the intervention studies that used various art forms for training in palliative care, their conclusions cannot be validated because they did not report all the literature used. Ike and Howell’s ^20^ review discussed only the validity of the psychometric scales or quantitative observation metrics used in intervention studies. Although Perry *et al.,* ^21^ Mukunda *et al.,* ^22^ and Alkhaifi *et al.* ^23^ concluded that such studies offer reasonable evidence in favor of using visual arts to improve clinical observation skills in medical education, they did not sufficiently scrutinize how the included studies defined and measured observational skills. Additionally, Mehta and Agius ^24^ focused on diagnostic skills in medical education. It is argued that the interventions’ effect on diagnostic abilities has insufficient evidence as only one study has been investigated, and we should examine whether the skills gained are transferable to clinical practice.

Thus, the present article maps the intervention studies that used visual art observation to develop observation skills for novice healthcare learners, namely, students, residents, trainees, or inexperienced workers in related majors or domains. It also scrutinizes what they measured as observational skills and discusses their effects on such technical skills that the advocates argue.

Therefore, this review will answer the following research questions (RQs): 1) What key features or trends characterize visual art observation training in intervention studies aimed at improving observation skills? 2) How did the studies measure the outcome related to observation skills, and what results were obtained? 3) Do the observational skills that the intervention studies intend to foster or measure lead to expert performance? 4) What are the recommended methods and critical issues in future research?

The following sections of our paper are structured as follows. The Methods section explains the process of this scoping review. The Results section overviews the intervention studies and answers RQ1 and RQ2. The Discussion section discusses the review results regarding RQ3 and presents limitations and implications for future research as an answer for RQ4. Finally, we present the study’s main conclusions.

## Methods

### Search strategy and selection criteria

I designed a scoping review protocol following the PRISMA Extension for Scoping Reviews (PRISMA-ScR). ^25^

As a preliminary search to plan the search strategy, I collected 12 relevant intervention studies ^14^^,^^26^^–^^36^ from previous review studies ^17^^–^^24^^,^^37^ with a manual search by the end of 2023. This result is a part of the published paper as version 1. ^38^

According to the result, studies were included if: (i) participants were novice healthcare learners such as undergraduate or post-graduate students, residents, and trainees in any health professional education; (ii) participants were enrolled in a visual art observation training program of any duration; (iii) the main objective of the study was to evaluate the effect of the intervention on technical skills related to observation; (iv) these skills were objectively measured, such as using psychometric scales, quantitative metrics, or grading participants’ statements by predetermined criteria; and (v) the main text was written in English.

On the other hand, studies were excluded if: (i) the full text could not be obtained; (ii) the main text was written in other than English; (iii) review articles; (iv) intervention studies that used visual art but do not aim to develop observation skills; (v) interventions included other activities, except for pre- and posttests, such as drawing or creating visual arts and observing medical images; (vi) studies on “graphic medicine,” ^39^ that used comics included written dialogues; (vii) outcome measures for the skills were only subjective measures, irrespective of the method used (e.g., participants’ subjective evaluation of their skills, their free comments on the interventions, and scales of their impressions of the interventions), (viii) studies that extracted key concepts from the participants’ comments, such as thematic analysis, ^40^ because they are not suitable for determining the learning effects for the entire concerned population since the concepts are identified from the comments of only a few participants, or (iv) the participants were not novice healthcare learners, or it is not clear that they were.

To confirm the rigor of the selection process, the reference lists of included studies or relevant reviews identified through the search were hand-searched to identify other relevant papers. Moreover, by comparing the results of the preliminary search, any missing studies were included in the final search result.

### Data collection

The search included publications from MEDLINE, ERIC, APA PsycInfo, and Open Dissertations in EBSCOhost published since 2001, the year the seminal paper by Dolev et al. ^27^ was published. This search was undertaken on January 16, 2025. The search terms ( Table 1) were determined by including as many of the titles of the papers collected in the preliminary search and included in Mehta and Agius’s ^24^ recent scoping review as possible while also considering a balance that would not overload the search results through repeating trial searches. Along with the search terms, some subject terms were set as exclusion parameters, as mentioned in Table 1. After these databases were searched separately, an integrated search across these databases was conducted to exclude duplicate records automatically. It was desirable to include CINAHL, but it was impossible because my institution does not subscribe to this database.

**Table 1.** Search terms entered into the databases.

TI (((art OR arts OR “visual thinking” OR “visual literacy” OR “visual art*” OR (observati* AND skill*)) AND (train* OR student* OR resident* OR educati*)) NOT (“art therapy” OR “art education” OR “arts education” OR “state of the art” OR “state-of-the-art” OR “the art and science”) OR ((art OR visual) AND (diagnostic OR perception) AND (train* OR student* OR resident* OR educati*)) OR (((art OR arts) AND observ* AND patient*) NOT (“art therapy” OR “art education” OR “arts education”))) NOT SU (“academic achievement” OR “language arts education” OR “art therapy” OR “middle school students” OR “high school students” OR “elementary education” OR “elementary secondary education” OR “ secondary education” OR “art teachers” OR “preservice teacher education” OR “elementary school student” OR “childhood education” OR “teacher education” OR “elementary school students” OR “special education” OR “science education” OR drawing OR “creative arts therapy” OR music OR “physical education” OR “career development” OR “middle school teachers” OR dance OR drama)

### Data analysis

All the records retrieved from searches were loaded into Excel. Duplicates that could not be removed automatically were removed manually. Then, I screened titles, abstracts, and full-text articles for eligibility. I extracted relevant information on the research questions from the papers of each included study without additional inquiry into the study authors. The extracted data was illustrated in a tabular and descriptive format, linking it to the research questions.

## Results

**Figure 1.**
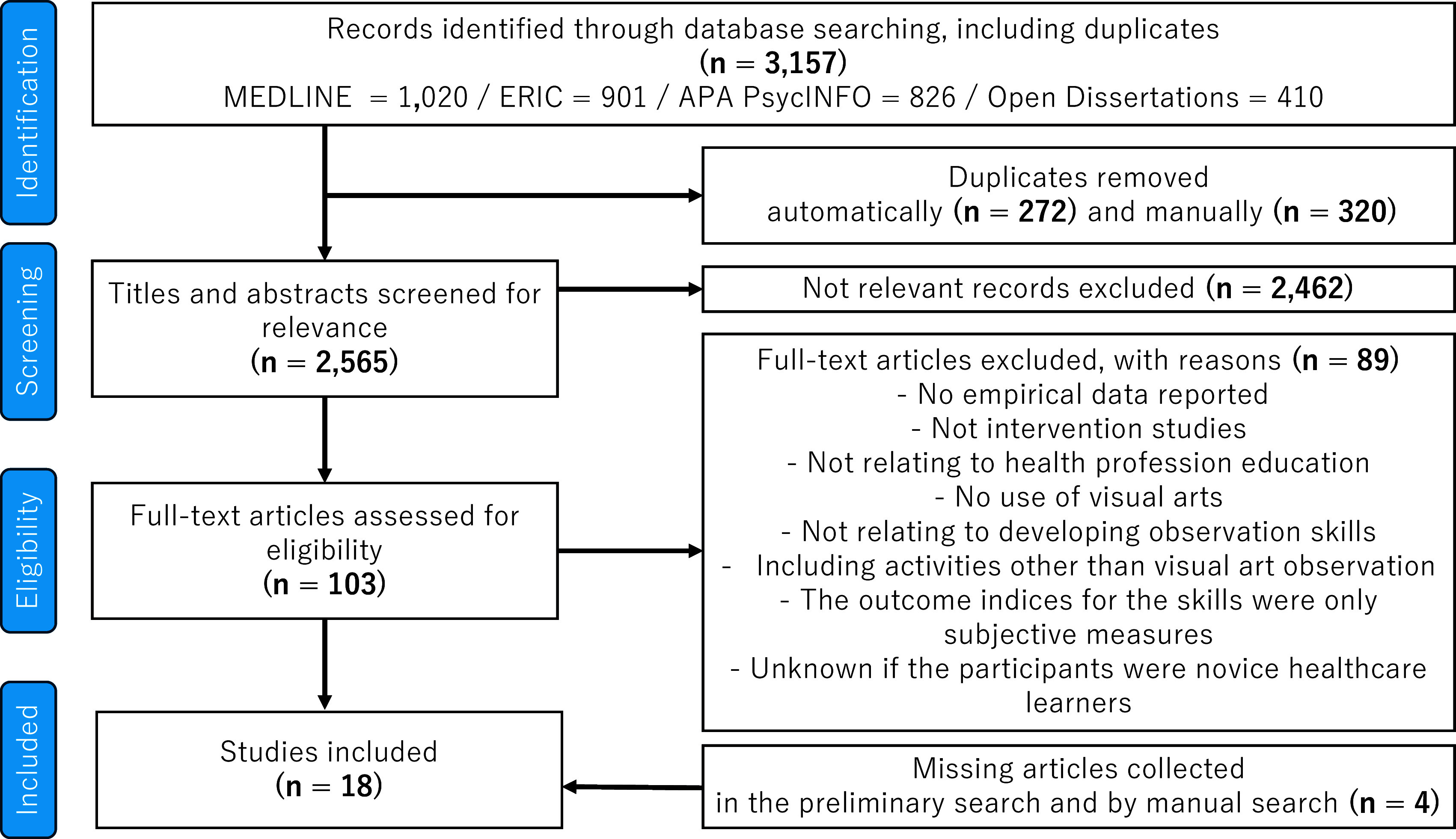
Flow diagram for the scoping review process.

I identified 3,157 publications, of which 18 articles ^14^^,^^26^^–^^31^^,^^33^^–^^36^^,^^41^^–^^47^ were included (See Figure 1 and Appendix Table (Underlying data)). The 18 articles included three studies in veterinary education. They were comparable to training and experimental designs in human health professional education.

I will describe the results in line with RQs.

**RQ1) What key features or trends characterize visual art observation training in intervention studies aimed at improving observation skills?**

### Goals

Though three of these intervention studies stated their goals simply such as “enhancing observation skills,” the majority targeted specific skills regarding observation skills, such as perception, interpretation, description, emotional recognition, reflective thinking, tolerance for ambiguity, skills or interest of communication, empathic skills, and collaboration with team members. Additionally, Agarwal *et al*. ^26^ aimed to increase the time participants spent analyzing a clinical image, the length of their response, and the degree of clinically relevant descriptive content. Ho *et al.* ^45^ aimed to enhance visual literacy, namely, the ability to discriminate and use visual cues to communicate with others. Greige *et al.* ^44^ aimed to introduce to the world of visual arts. Moreover, two studies (Gurwin *et al.* ^31^ and Wolf *et al.* ^47^) aimed to develop observation skills specific to each expertise.

### Participants

The intervention studies were conducted for undergraduate or post-graduate students, residents, and trainees in various disciplines, such as radiology, dermatology, nursing, neurology, ophthalmology, and veterinary medicine. Although they were implemented in multiple grade levels and programs, none were multicenter studies. Twelve were done in the United States, including four studies at Yale University. Other studies took place in the United Kingdom, Italy, India, and Canada. Sample sizes in these studies were relatively small, from 7 to no more than 101.

### Sessions

Sessions in these interventions were held in both required and elective curricula courses and extracurricular activities. The frequency and duration of sessions in each study ranged from a single session lasting only a few hours to multiple sessions over two months. Eleven included activities in art museums.

### Main activities/Protocol

In all the interventions, the participants described the images’ details. In addition, they often discussed their interpretations. Fifteen studies utilized the discussion involved in or among the participants. The remaining three studies did not provide the information, but some could be omitted.

Some interventions utilized or applied established and well-known teaching programs. Six studies utilized Visual Thinking Strategies (VTS). VTS has basic tenets that must be obeyed: using the three core questions, paraphrasing with conditional language, linking, framing, resisting the impulse to insert facilitators’ opinions into the discussion, and a non-summative conclusion. ^12^ The core questions are: “What’s going on in this picture?” “What do you see that makes you say that?” and “What more can we find?” These questions could help participants to observe the entire painting carefully and make inferences based on the visual evidence. ^48^ Jasani and Saks ^33^ adapted VTS to the 4-step method, consisting of observation, interpretation, reflection, and communication. Gurwin et al. ^31^ utilized the Artful Thinking approach, which consists of Observing & Describing, Questioning & Investigating, Reasoning, Comparing & Connecting, Exploring Viewpoints, and Finding Complexity. ^49^ However, the authors did not seem to use Finding Complexity.

Moreover, ten studies instructed or clearly encouraged the participants to examine the whole image in detail. Thirteen interventions, including ones using VTS, focused on or highlighted, separating the description of visual evidence from the interpretation. Four taught the participants the concepts of visual elements, such as color, light, and texture.

On the other hand, the studies seldom reported providing a de-/brief on the relationship between visual art observation and healthcare practice, though Ker *et al*. ^12^ recommends such de-/brief. It is possible they forgot to report this information.

### Types and numbers of visual arts used

Among the interventions that provided related information, they often used paintings, including their reproduced images, sketches, photographs, sculptures, and ancient coins. In the choice, protocols such as VTS and the goals of the interventions tend to be considered. Because the number of sessions varied among the studies, the number of visual arts used also varied, though nine studies did not report the number.

**RQ2) How did the studies measure the outcome related to observation skills, and what results were obtained?**

### Experimental designs

Eighteen studies were included in this study. Four included studies were randomized controlled studies with pre- and posttests. One study was a randomized controlled study with posttests only. Two were not randomized controlled studies with both tests. The study was not a randomized controlled study with posttests only. Ten were not controlled studies with both tests.

### Timing of the pre- and posttests

In the included studies, pretests were often administered just before the sessions, and posttests were done at the end or within one week after the sessions ended. At the same time, eight studies did not provide this information. Fernandez *et al.* (2021) ^41^ and Fernandez *et al.* (2022) ^42^ had participants undergo cytology instruction or laboratory training between the immediate and delayed posttests.

### Statistical analysis and tests

Many studies misused statistical tests to interpret their results. Twelve did *t*-tests for non-randomized samples. This does not meet their prerequisite, randomized samples. Agarwal *et al*. ^26^ reported that they analyzed variance (ANOVA) but seemed to conduct *t*-tests repeatedly for all the pairs inappropriately, as far as the test results they showed. Gurwin *et al.* ^31^ also should use an ANOVA instead of *t*-tests for the pre-and posttest increments.

### Outcome measures and their results

As measures for observation skills, all the intervention studies used visual images: eight used clinical and artistic, nine clinical only, and one artistic only. The number of images used in each test varies widely, from two to 15, among the studies that stated the information.

In the tests, the participants were usually asked to describe visual features in the images. What features to write about varied. For artistic images, five studies were asked to write what they observed, two were asked to write about the VTS core questions, and a study was asked to spot the differences. For clinical images, the participants were asked to write freely about their observations and write down as many (abnormal) signs as possible, identify them, and write their (clinical) interpretations. Griffin *et al*. ^29^ asked participants to identify key visual features and comment on visual elements such as texture, lines and contour, color (or shading), and contrast for both images.

Of the 16 studies that conducted pre- and posttests, one used the same images in both tests, twelve used different images between the tests, and three did not report the information. If images differ between the tests, procedures should be included to equalize their difficulty. As the equalization, three of the 12 studies randomly assigned one of the two versions as the pretest and the other as the posttest. Ho *et al.* ^45^ reported that similar complexity between the tests was confirmed by inter- and intra-assessment analysis but did not provide the details or data. In the other (8) studies, the authors assessed the difficulties similarly but did not provide evidence, while others did not offer sufficient information. Indeed, the results of Fernandez *et al.* (2022), ^42^ which differed from theoretical predictions, are suspected to be due to a lack of such equalization.

The scoring criteria by which the studies quantitatively or qualitatively evaluated their descriptions varied. In analyzing the criteria, Greige *et al.* ^44^ is excluded due to serious suspicions of inconsistencies in the points allocated to each task between the tests, the use of a rubric mixed with multiple perspectives, and unknown details of the rubric. Snigdha *et al.* ^46^ is also excluded because only the composite score is shown, and it is not explained how each task was scored and how each score was composited.

Then, we examined the validity of the results for each criterion with the following principles:

■ The results, with inappropriate use of statistical tests, are adopted, but the results of the tests are ignored, and only the measured values are considered.
■ The results with problems in testing procedures are not adopted, specifically when reasonable procedures to equalize difficulty cannot be confirmed, such as when different images were used between tests or when it is unknown whether the same or different images were used in each test.
■ Priority is given to results from the studies that had valid experimental designs.
■ The criteria in each study are categorized based on their qualitative similarity, though not in a strict manner, as the images used in the testing differ across the studies.
■ Because the included studies are not multicenter, only results from three or more studies are considered except for those not adopted in each category.
■ Only result trends are considered, i.e., positive or negative, as the criteria scales also vary between the studies.

Table 2 summarizes the scoring criteria and their results for artistic images. Roughly classified, the categories are Describing more elements, Using more words, Using more subject words, Using more technical vocabulary, Critical thinking, and Other thinking skills. In Describing more elements, positive results tend to be more than negative ones. None of the others have enough studies to consider.

**Table 2.** The scoring criteria and their results for artistic images.

Category	Criteria	Results	Studies	Control experiments	Both pre- and posttest	Notes
Describing more elements	Number of elements identified (marked with the rubric)	Significantly higher for both immediate and delayed posttest than for pre-test	Fernandez *et al.* (2021) ^41^	No	Yes	D,S
	Number of objective observations (rated by experts)	C: no significant change. Iv: significant increase in the delayed posttest compared with the immediate posttest	Fernandez *et al.* (2022) ^42^	Yes	Yes	D
	Number of accurate observations (marked with the expert rubric)	For both groups, significantly improved from the pre-test to the immediate posttest and delayed posttest	Fernandez *et al.* (2022) ^42^	Yes	Yes	D
	Number of elements identified (in an answer to the VTS core questions, but unknown the detail of the scoring)	Iv>C significantly in the increment	Ferrara *et al.* ^43^	R	Yes	D
	Observation skill: identifying the figures/objects and the details in the background of the scene (in answer to the VTS core questions, marking with the rubric)	Iv>C significantly in the increment	Ferrara *et al.* ^43^	R	Yes	D
	Frequency of descriptions of key visual features and visual elements (preset by an arts educator)	Iv>C, but not significant	Griffin *et al.* ^29^	NR	No	S
	Frequency of single factual declaration	Significantly increased	Klugman *et al.* ^34^	No	Yes	S
	Frequency of descriptions of names or identifying something (as an answer to the VTS core questions)	Increased, but not significant	Poirier *et al.* ^36^	No	Yes	S
	Frequency of descriptions of a single factual declaration (in an answer to the VTS core questions)	Increased, but not significant	Poirier *et al.* ^36^	No	Yes	S
Using more words	Number of words used for the description	No significant difference between the groups/Iv increased significantly from pre-test to immediate and delayed posttest	Fernandez *et al.* (2022) ^42^	Yes	Yes	D
	Number of words used for the description	Significantly increased	Klugman *et al.* ^34^	No	Yes	S
	Number of words used for the description (in an answer to the VTS core questions)	Iv>C, but not significantly, in the increment	Ferrara *et al.* ^43^	R	Yes	D
Using more subject words	Number of subjective observations (rated by experts)	No significant change over time	Fernandez *et al.* (2022) ^42^	Yes	Yes	D
Using more technical vocabulary	Number of specific (technical) vocabulary	Both groups demonstrated significant increases on the delayed posttest compared with both the pre-test and the immediate posttest	Fernandez *et al.* (2022) ^42^	Yes	Yes	D
Critical thinking	Critical thinking, describing multiple hypothetical interpretations of the scene considering corresponding supporting reasonings (in answer to the VTS core questions, marking with the rubric)	Iv>C significantly in the increment	Ferrara *et al.* ^43^	R	Yes	D
	Frequency of using terms related to critical thinking skill	Iv>C significantly in the increments	Gurwin *et al.* ^31^	R	Yes	D,S
Other thinking skills	Linguistic expression, elaborating a narrative that deals with characters, dynamics, emotions, and relationships present in the scene in accordance to the type of image (in answer to the VTS core questions, marking with the rubric)	Iv>C significantly in the increment	Ferrara *et al.* ^43^	R	Yes	D
	Problem-solving, considering the scene as a whole and the relationships among elements, and encompassing empirical details, personal experience, and prior knowledge (in answer to the VTS core questions, marking with the rubric)	Iv>C significantly in the increment	Ferrara *et al.* ^43^	R	Yes	D

Abbreviations: VTS: Visual Thinking Strategies; R: a randomized controlled study; NR: not-randomized controlled study; S: the inappropriate use of statistical tests or analysis, mentioned in the text; D: any reasonable procedures to equalize the difficulty cannot be confirmed when different images were used between tests or unknown whether the same or different images were used in each test; Iv: the intervention group; C, the control group.

Table 3 also summarizes clinical images. Roughly classified, the categories are Using more words, Using more subjective words, Using more technical vocabulary, Describing more signs, Describing more visual elements, Signs identified, Diagnostic comments, Observation of patients and their contexts, Speculative thinking, Looking longer, and Others, while there are some unclassifiable. In Describing more signs, positive results tend to be more than negative ones. None of the others have enough studies to consider.

**Table 3.** The scoring criteria and their results for clinical images.

Category	Criteria	Results	Studies	Control experiments	Both pre-and posttest	Notes
Using more words	Number of words used for the description	Iv: significantly increased, C: increased, but no significant	Agarwal *et al.* ^26^	NR	Yes	D,S
	Number of words used for the description	There is no significant difference between groups, and over time	Fernandez *et al.* (2022) ^42^	Yes	Yes	D
	Number of words used for the description	Significantly increased	Klugman *et al.* ^34^	No	Yes	S
	Number of words used for the description	Iv>C, significantly in 5 out of the 6 photos	Pellico *et al.* ^35^	R (probably)	No	
	Number of words used for the description	Increased for radiographs, but not chart	Wolf *et al.* ^47^	No	Yes	D
	Number of words used for the description (in an answer to the VTS core questions)	Iv>C, but not significantly, in the increment	Ferrara *et al.* ^43^	R	Yes	D
Using more subjective words	Number of subjective observations (rated by experts)	No significant change over time in either group	Fernandez *et al. (*2022) ^42^	Yes	Yes	D
	Frequency of use of subjective terminology	Decreased	Jasani and Saks ^33^	No	Yes	D
Using more technical vocabulary	Number of specific (technical) vocabulary	Iv increased significantly, but not until the delayed posttest. C increased significantly from pretest to the immediate and delayed posttest	Fernandez *et al.* (2022) ^42^	Yes	Yes	D
Describing more signs	Frequency of clinical observations	Iv: significantly increased, C: increased, but no significant	Agarwal *et al.* ^26^	NR	Yes	D,S
	Frequency of describing visual diagnostic features (marked by a key)	Iv’s posttest score significantly improved by 56%, and it was 10-12% higher than that of the other groups	Dolev *et al.* (intervention between 1998 and 1999) ^27^	R	Yes	
	Number of accurate observations (marked with the expert rubric)	Significantly improved from the pretest to the immediate posttest for both groups. For the delayed posttest, C decreased significantly to pretest levels, while Iv did not change significantly from the immediate to the delayed posttest	Fernandez *et al*. (2022) ^42^	Yes	Yes	D
	Frequency of describing visual diagnostic features (marked by a key)	Improved, but no significant	Garino (intervention in 2009) ^28^	No	Yes	S
	Frequency of unique observations	Increased, but no significant	Jasani and Saks ^33^	No	Yes	D,S
	Frequency of single factual declaration	Significantly increased	Klugman *et al.* ^34^	No	Yes	S
	Frequency of plausible objective clinical findings	Iv>C, significantly in 5 out of the 6 photos	Pellico *et al.* ^35^	R (probably)	No	
Describing more visual elements	Number of objective observations (rated by experts)	C: No significant change over time Iv: significant increase in the delayed posttest compared with both the pretest and the immediate posttest	Fernandez *et al. (*2022) ^42^	Yes	Yes	D
	Frequency of correct answers (key visual features and visual elements, pre-set by dermatologists)	Iv>C, but no significant	Griffin *et al.* ^29^	NR	No	S
Signs identified	Whether the abnormality was identified	Significantly improved	Goodman and Kelleher ^14^	No	Yes	D,S
Diagnostic comments	Frequency of diagnostic comments	Iv and C: decreased, but no significant	Agarwal *et al.* ^26^	NR	Yes	D,S
Observation of patients and their contexts	Frequency of general patient observations	Iv: significantly increased, C: increased, but no significant	Agarwal *et al.* ^26^	NR	Yes	D,S
	Linguistic expression, elaborating a narrative that deals with characters, dynamics, emotions, and relationships present in the scene in accordance to the type of image (in answer to the VTS core questions, marking with the rubric)	Iv>C significantly in the increment	Ferrara *et al.* ^43^	R	Yes	D
	Scope of interpretations involving the patient’s surroundings, the patient’s perspective, or emotional state	Increased	Jasani and Saks ^33^	No	Yes	D
	Frequency of describing elements other than people	Mixed, but C tended to be higher than Iv, and Iv<C significantly in 1 out of the 6 photos	Pellico *et al.* ^35^	R (probably)	No	
Speculative thinking	Critical thinking, describing multiple hypothetical interpretations of the scene considering corresponding supporting reasonings (in answer to the VTS core questions, marking with the rubric)	Iv>C, but not significantly, in the increment	Ferrara *et al.* ^43^	R	Yes	D
	Frequency of the words related to speculative thinking	Increased	Jasani and Saks ^33^	No	Yes	D
	Frequency of alternative diagnosis offered	Iv>C, significantly in 5 out of the 6 photos	Pellico *et al.* ^35^	R (probably)	No	
Looking longer	Time spent	Iv: increased, but no significant C: decreased, but no significant	Agarwal *et al.* ^26^	NR	Yes	D,S
Others	Frequency of “self-deprecating” remarks	Iv: increased, but no significant C: decreased, but no significant	Agarwal *et al.* ^26^	NR	Yes	D,S
	Problem-solving, considering the scene as a whole and the relationships among elements, and encompassing empirical details, personal experience, and prior knowledge (in an answer to the VTS core questions, marking with the rubric)	Iv>C significantly in the increment	Ferrara *et al.* ^43^	R	Yes	D
	Frequency of use of visual analogies	Increased	Jasani and Saks ^33^	No	Yes	D
Unclassifiable: Describing more elements, but unknown for signs or visual elements	Number of elements identified (marked with the rubric)	No significant difference between any of the tests (37.5%, 44.4%, and 36.8% for pretest, immediate posttest, and delayed posttest)	Fernandez *et al.* (2021) ^41^	No	Yes	D,S
	Number of elements identified (in an answer to the VTS core questions, but unknown the detail of the scoring)	Iv>C, but not significantly, in the increment	Ferrara *et al.* ^43^	R	Yes	D
	Observation skill: identifying the figures/objects and the details in the background of the scene (in answer to the VTS core questions, marking with the rubric)	Iv>C significantly in the increment	Ferrara *et al.* ^43^	R	Yes	D
	Total number of observations made (scoring procedure unknown)	Iv significantly increased. But posttest score was not significantly different between groups	Ho *et al.* ^45^	NR	Yes	D,S
Unclassifiable	Observations (marked by the key validated by three experts, but the detail unknown)	Posttest scores for Session 1 were significantly higher than pretest. Posttest scores for Session 2 were higher, but not significantly, than those of Session 1.	Grossman *et al.* ^30^	No	Yes	D,S
	Interpretations (marked by the key validated by three experts, but the detail unknown)	Posttest scores for Session 1 were significantly higher than pretest. Posttest scores for Session 2 were higher, but not significantly, than those of Session 1.	Grossman *et al.* ^30^	No	Yes	D,S
	The combined frequency of observations (marked by a key) with the general quality of the description (rated by experts and a medical student)	Iv>C significantly	Gurwin *et al.* ^31^	R	Yes	D,S
	Descriptive ability (scoring procedure unknown)	Iv significantly increased. But posttest scores was not significantly different between groups	Ho *et al.* ^45^	NR	Yes	D,S
	Observations (marking with the rubric *)	Significantly increased in radiographs. Increased, but not significantly, in the chart.	Wolf *et al.* ^47^	No	Yes	D,S
	Clinical interpretation (marking with the rubric *)	Significantly increased in radiographs. Increased, but not significantly, in the chart.	Wolf *et al.* ^47^	No	Yes	D,S

Abbreviations and marks: Same as Table 2.

* Due to a website error, the supplementary material describing the rubric could not be accessed.

In addition to using these images, Gurwin *et al.* ^31^ used the Reading the Mind in the Eyes test, a test for speculating the person's mind from images of their eyes. They reported no significant difference between the intervention group and the control group in the clement, due to the ceiling effect. Klugman *et al.* ^34^ used Budner’s Tolerance of Ambiguity Scale and the Communications Skills Attitudes Scale and reported a significantly positive change in both scales, although these scales are subjective.

Hence, few studies had valid and reliable experiments, although it must be quite difficult to execute such experimental designs in educational and training settings. The relatively valid evidence is that the participants’ descriptions of artistic or medical images revealed that participants were able to list more elements or signs. This seemed to be related to skills to capture and document clearly what is observed (in teamwork). On the other hand, there are not enough valid studies for the others of the technical skills that the intervention studies intended to foster: tolerating being unsure for long enough to sustain a meaningful analysis; assessing medical images and patient symptoms accurately to reduce misdiagnosis; metacognitive awareness; gleaning nonverbal cues to support how they care for a patient; understanding or extraction of the patient’s background and contexts; and perspective-taking. No studies also examined whether the participants could separate interpretation from description, though most included studies intended to teach to be.

## Discussion

In this section, we discuss the results of this review to answer RQ3, do the observational skills that the intervention studies intend to foster or measure lead to expert performance?

### Listing more elements of artistic images

Generally, expert's technical skills for observation do not transfer to images outside of his or her expertise. Experimental studies showed that in terms of radiology, expertise was positively transferred to the task of finding small low-contrast dots in phantom X-ray images ^50^ but not to the task of finding hidden targets in pictorial scenes. ^51^ The same can be said for visual recollection memory. Expert cytologists and radiologists were not better at recognizing scenes or isolated objects than non-medical participants. ^52^

Thus, even if the interventions can facilitate listing more elements of artistic images, the transfer to clinical images needs to be investigated. No included studies did, although some studies examined artistic and medical images. Detecting differences in non-clinical images, such as those used by Snigdha *et al*., ^46^ may not be useful.

### Longer periods of careful and systematic observations for accurate diagnosis

Listing more signs for medical images, as well as spending more time as Agarwal *et al*. ^26^ demonstrated, indicates observing carefully and thoroughly.

However, encouraging novice medical learners to look all over does not lead to an accurate diagnosis. In Kok *et al.*’s study for medical students, ^53^ systematic viewing intervention, such as detecting radiograph abnormalities by viewing the images in a given order, or full-coverage viewing intervention, such as mentally dividing each image into nine imaginary segments (3×3) and inspecting each segment separately, did not outperform non-systematic viewing intervention, wherein participants were urged to inspect whatever attracted their attention, notwithstanding that systematic and full-coverage viewing took a longer (but not significant) time than non-systematic viewing. Van Geel *et al.* ^54^ also reported similar results.

In addition, observing for a longer period is unnecessary for an accurate diagnosis. Experts take less time than novices to diagnose accurately. In contrast, they are more likely to misdiagnose if they take longer. ^55^^–^^57^

Moreover, observing and describing more visual features or signs in medical images or patient photographs do not lead to accurate diagnoses for novices. Novices, such as medical school students, generate hypotheses based on constant information input and cannot select appropriate hypotheses. ^58^ An experiment that presented electrocardiograms to non-medical laypersons revealed that participants usually misdiagnosed the problem because they failed to eliminate irrelevant features. ^59^ The same can be said for experts. Expert general practitioners can diagnose correctly without discovering all the disease features. ^60^ That is because experts can extract more helpful information about the given situation, immediately generate appropriate hypotheses, eliminate irrelevant hypotheses, and test the hypotheses. ^61^^–^^63^

In sum, it is important to find critical evidence, such as signs, to distinguish a specific disease from other possibilities for an accurate diagnosis by looking at medical images and patient interviews. The effect of visual art observation on such discrimination needs to be investigated, though no included studies have done so.

For the same reasons, tolerance of ambiguity may lead to observing thoroughly but not to diagnostic accuracy. Indeed, Klugman et al. ^34^ reported a significant positive change in Budner’s Tolerance of Ambiguity Scale but did not demonstrate that tolerance promoted such observation. It is also necessary to investigate how tolerance as a psychological factor works in healthcare practice. This is because it may not be a trait but rather a state, i.e., susceptible to environmental changes, given the findings on misdiagnosis. Misdiagnosis causes are complex, ^64^ including environmental factors, ^65^ and may not always be due to mere carelessness or lack of tolerance. Besides, experts’ mental images can diagnose quickly and accurately and are compromises between fitting the data and minimizing the complexity of the model, ^66^ which leads to experts’ perceptions toward certain classes of stimuli in the domain. ^67^ That many expert radiologists overlook the gorilla inserted into chest CTs ^68^ demonstrates that experts’ errors, such as premature closure, may be a byproduct of the development of technical ability. ^67^^,^^69^

### Technical knowledge and skills based on observation skills

Cultivating mental images based on technical knowledge and skills is needed to find critical evidence to distinguish a specific disease. Many studies ^55^^,^^70^^–^^72^ and reviews ^73^^–^^79^ on medical image perception have shown that experts’ diagnosis is generally characterized by holistic and quick recognition. This is due to pattern recognition ^60^^,^^80^^,^^81^ or intuition composed of scripts, schemas, or mental models/images/representations (hereinafter collectively referred to as mental images). ^61^^,^^82^ Experts compare medical images or patients with mental images—associated with domain-specific knowledge—of normal and abnormal cases at a glance. ^62^^,^^67^^,^^83^^,^^84^ A study using fMRI ^85^ revealed that when radiologists looked at radiographs, the brain regions related to visual attention and the encoding, storing, and retrieval of visual memory were activated.

Humans generally recognize things by associating them with mental images formed through our daily visual experiences, not just medical images. ^66^ For example, humans can recognize a scene’s gist and gaze into areas of interest through their knowledge of the world. ^86^^–^^88^ This top-down control of gaze using mental images is common for humans. Likewise, the experts’ intuition is cultivated through the accumulation of domain-specific knowledge and experiences. Indeed, the number of mammograms observed is proportional to the accuracy of the diagnosis. ^89^ On the other hand, in the context of novices’ misdiagnoses, Brunyé *et al.*’s ^90^ review showed that search errors generally occur when novices overlook definitive features with subtle visual features and repeatedly look at areas that are irrelevant for a diagnosis. Misdiagnoses also happen when signs of extremely low prevalence are overlooked by novices (and experts). Novices will also likely make recognition errors due to insufficient mental images and knowledge. ^70^^,^^84^ This implies that novices’ errors are caused by the lack of top-down gaze control due to inadequate mental images.

Thus, it is critical to consider the participants’ technical knowledge and skills as a mediator variable in measuring observation skills, though no included studies did.

### Conscious and unconscious observation

The intervention studies emphasize conscious observation but do not seem to consider the unconscious process such as tacit knowledge ^91^ and intuition. For example, a theory of perceptual learning highlights the unconscious learning process in pattern recognition. It is possible even if the rationale of such recognition is not verbalized, although it is more effective if it is verbalized. ^92^ Experimental psychology has shown that simply looking at various paintings with the artists' names randomly can enable novices to differentiate between artists. ^93^^–^^95^ Similar results were obtained in experiments that had novices diagnose psychopathological cases presented visually (through words) or aurally. ^96^ Additionally, novices can detect melanoma by showing benign and malignant cases side by side, even without instructions, such as the ABCD rule. ^97^ In contrast, instructing novices on such rules has little effect on diagnostic accuracy. ^97^^,^^98^

Learning modules based on theories of perceptual learning, such as Perceptual and Adaptive Learning Modules (PALM), have been developed to foster pattern recognition of medical images. ^81^^,^^99^ A complete discussion of them is beyond the scope of this article; all the relevant studies that we gathered have reported excellent results of diagnostic accuracy for medical school students and/or residents ^100^^–^^107^ and non-medical participants, ^108^^–^^111^ however, it is possible such excellent results could be due to publication bias. Guégan *et al.*’s review ^112^ also concluded that the modules are promising educational tools to improve diagnostic accuracy and rapidity in daily medical practice. Notably, the outcome measures used by these modules included fluency or rapidity of recognition. This is consistent with research findings on medical experts’ performance mentioned above. Therefore, PALM may be an alternative to visual art observation and is worthy of conducting comparative studies.

### Fostering verbalizing skills for communication with patients and teamwork

Listed more elements or signs may be linked to skills of verbalizing what one observes, even if it does not help in making an accurate diagnosis. It is assumed that such skills could be useful for patient communication and teamwork. However, the intervention studies have not provided sufficient evidence that listing more elements or signs contributes to such communication. Also, the included studies sometimes taught the participants the concepts of visual elements by using visual arts. Still, they did not explain or demonstrate how the concepts could be used in healthcare practice.

Instead, it may not be visual art observation that contributed to the verbalizing skills, but discussions accompanied it. However, the intervention studies did not examine the impact of the talks separating from that of visual art observation, nor did they describe what instructions and advice were given to the participants for enhancing the quality of the group discussions, except for Jasani and Saks ^33^ and Klugman *et al.* ^34^ that instructed them to think collaboratively and share and combine each other’s ideas.

If discussions contribute to verbalizing skills, finding a rationale for visual art observation for cultivating the skills may be difficult. That is because there could be methods directly related to healthcare practice without being bypassed by visual art observation. Such methods include observing and discussing medical images using the three core questions of the VTS, as well as drama, theater-based improvisation, or role-playing in clinical situations. ^113^ Indeed, Choe *et al.* ^114^ demonstrated that teaching art formal analysis, i.e., analyzing through the concepts of visual elements, of radiologic images, NOT visual arts, improved not only descriptions but also diagnoses of mammographic images and chest radiographs.

### Long-term effects such as scaffolding

The long-term effects of visual art observation are unknown because no included studies were examined. Likewise, it is worth investigating whether visual art observation is scaffolding for further learning in technical education and training. Fernandez *et al.* (2021) ^41^ and Fernandez *et al.* (2022) ^42^ conducted a visual art observation training followed by technical skills lessons. If this is true, learners who take visual art observation interventions should be able to acquire technical knowledge and skills faster or more deeply than those who do not. To investigate that, longitudinal studies are required ^17^ because the effects of such scaffolding may be delayed.

### Limitations

This article has several limitations. First, I excluded articles published before 2001 or written in a language other than English and those that subjectively measured observation skills or included activities other than visual art observation. In addition, there might be studies that should be included in the CINAHL database that we could not search. These studies may have included information that could have contributed to this article.

Second, it is possible that the selection of included studies was skewed because only I judged to in-/exclude and set many search criteria in the database search to make the volume of data manageable. However, this skew may be compensated for to some degree by manually searching the reference lists of the articles obtained through the search process.

Third, since all included intervention studies were published in academic journals, publication bias could lead to overestimating their effects.

Fourth, there may also be bias due to inadequate disclosure of information about intervention methods and outcome measures in the included studies.

Fifth, I excluded intervention studies using visual arts to foster technical skills other than observation skills. Then, the intervention studies may examine the observation-related skills that the included intervention studies rarely did. Other reviews are needed to discuss these studies.

Finally, in health professional education for both novices (pre-service) and experts (in-service), the arts can play many roles beyond enhancing observational skills. ^115^^–^^122^ However, discussing such roles is outside the scope of this article. The author does not intend to extrapolate the conclusions to technical skills beyond those related to observation, nor the role of the arts in health professional education.

### Implications for future research

Because the lack of sound evidence precludes offering suggestions regarding the practice of visual art observation, our discussion focuses on the implications for future research regarding RQ4.

We also fully agree with Mehta and Agius’s suggestions, ^24^ such as conducting a multicenter study, standardization in evaluating skill improvement across the studies, assessing long-term retention of skills, and determining whether the skills gained are transferable to clinical practice.

In addition to their suggestions, we would like to elaborate on some points. First, the participants’ technical knowledge and skills should be mediator variables in measuring observation skills. This is more important for longer intervention sessions or measuring long-term effects because novice healthcare learners concurrently should learn the knowledge and skills through education and training.

Second, it is strongly recommended that relationships between variables as outcome measures are examined. If the included studies had done so, they would have provided evidence for some of the issues addressed in the Discussion section. For example, they could have utilized their data to examine the correlation between descriptions of artistic and clinical images as well as the correlation between word count, length of time spent looking at images, or tolerance for ambiguity and quality of descriptions and interpretation or accuracy of diagnosis.

Third, it needs to investigate technical skills intended to be fostered and have rarely been studied, such as separating interpretation from description, tolerating being unsure for long enough to sustain a meaningful analysis, metacognitive awareness, perspective-taking, and gleaning nonverbal cues. It is also important to examine how visual elements can be integrated into healthcare practice.

Fourth, it is worth investigating how verbalizing skills fostered through visual art observation affect other technical skills or healthcare practices. For example, it may enhance self-reflection skills that require the use of language. It may also help to communicate with patients and colleagues.

Fifth, it is worth examining scaffolding for further learning in technical education and training. For example, to speak of accurate diagnoses, it might be an idea to investigate whether visual art observation is scaffolding for finding critical evidence to distinguish a specific disease.

Sixth, more careful consideration needs to be given to procedures for tests to measure outcomes. If the images differ between tests, the difficulties need to be equalized. Also, given that some intervention participants had negative inclement scores in the tests with an artistic and clinical image in Ferrara *et al.*, ^43^ tests with a few images appear to increase measurement errors and skew the results. On the other hand, comparisons using the same images across tests or studies could also be of value. Then, it might be good to use sufficient images, including both the same and different images.

Seventh, to measure the effectiveness of visual arts observation, it is recommended to use the same tests for measuring the effectiveness of learning modules on perceptual learning. Diagnostic accuracy and fluency as indicators of technical observation skills should also be examined, as asserted by Kellman *et al.* ^100^

Eighth, as Perry *et al.* ^21^ mentioned, the intervention methods must also be reported adequately. It is unlikely that merely looking at visual art develops technical skills. Instead, the design of the training, such as the rationale for the selection of the paintings, the type and number of paintings that have been viewed, de-/briefs on the relationship between visual art observation and healthcare practice, and instructions and facilitations for learners, including ways of reflections ^123^ and discussions, is required. Concerning studies using VTS, it is not enough to explain the three core questions; it is necessary to describe how the precise tenets of VTS were carried out since Ker *et al.* pointed out that some of the prior interventions deviated from the tenets. ^12^ It is also necessary to mention the learners’ responses evoked by the artworks, prompts, and questions to identify whether the outcomes were intended or derived. In addition, educational or training curricula and proficiency of the learner's technical knowledge and skills related to the aims and contents of the intervention should also be stated in detail because merely describing the learners' grades and training levels is insufficient for international readers to understand that. Moreover, I found the following deficiencies in the studies used in this article: the number of missing samples from the participants; what did the control group do; details of the content and format of the lectures that the control participants were subjected to; maximum possible values of the scales or items for considering the ceiling effect; details of the scoring criteria; how many schools/organizations did the participants belong to; how many images were used for the test; and timing of the tests. Thus, items listed in Appendix Table should be reported in future studies.

Ninth, research should directly compare promising interventions other than visual art observation, such as learning modules applied by perceptual learning and discussion using art formal analysis ^114^ of clinical images. For diagnosing medical images, teaching a specific eye movement search pattern that corresponds with the characteristics of the area shown in the radiographs ^124^ or demonstrating an expert model that searches visually and interprets symptoms (eye-movement modeling examples) ^125^ could also be effective. Performing arts such as drama could be beneficial for teamwork and communication skill development. ^113^ Due to time constraints imposed by the many demands of health professional education, it is necessary to identify a more efficient method among those available.

Finally, as Osman *et al.* ^126^ suggested, further research exploring the learning process through visual arts observation is required, as extant literature only focuses on outcomes. Dominant research methods such as controlled studies and psychometric scales do not allow for such investigation ^118^^,^^123^; thus, further research should include alternative methods and outcome measurements, such as qualitative methods. ^21^^,^^127^

## Conclusions

This scoping review mapped intervention studies using visual art observation for novice healthcare learners to foster observation skills. It scrutinized what they measured as observational skills and discussed their effects on technical skills. This review concludes that sound evidence is lacking for all the technical skills intended to be fostered, although some prior reviews concluded that such studies offer reasonable evidence. The intervention studies provided relatively valid evidence that the participants listed more elements or signs for artistic or medical images. However, observation skills for artistic images have not been demonstrated to transfer to technical skills. Nor can it be said that the evidence provided by the studies showed that they promoted accurate diagnoses and reduced misdiagnoses, as it contradicts the findings on novice and expert observational skills.

Additionally, the evidence on verbalizing skills is not isolated from the impact of discussions and is unclear regarding its transfer to actual communication. For the others, the technical skills, such as separating interpretation from description, tolerating being unsure for long enough to sustain a meaningful analysis, metacognitive awareness, perspective-taking, gleaning nonverbal cues, and understanding or extraction of the patient’s background and contexts, there are not enough valid studies. This is true for studies that directly examine promoting accurate diagnosis or reducing misdiagnosis. Moreover, there may be promising alternatives to visual art observation for cultivating such technical skills, but no comparative studies were conducted.

## Data Availability

OSF: Effects of visual art observation on technical skills in novice healthcare learners: A scoping review, https://doi.org/10.17605/OSF.IO/TDGQZ. ^128^ This project contains the following underlying data:
• Appendix Table.ods (the selected intervention studies' features, research design, and key results) Data are available under the terms of the Creative Commons Attribution 4.0 International license (CC-BY 4.0).
