# Supplementary material for: Effects of visual art observation on technical skills in novice healthcare learners: A scoping review
Source: F1000Res. 2026 Jan 24;12:79. Originally published 2023 Jan 19. [Version 3] doi: 10.12688/f1000research.129219.3 (PMC12759281; doi:10.12688/f1000research.129219.3)
Supplement: Peer review history

# Reviewer report: Reviewer response for version 3

**DOI:** 10.5256/f1000research.195407.r452893

**Published:** 2026-02-03

**Authors:** Sven Dupré

**Affiliations:** 1 Utrecht University, Utrecht, The Netherlands

The author addressed the issues raised in my review in an adequate way. I have no further issues,

Is the work clearly and accurately presented and does it cite the current literature?

Partly

If applicable, is the statistical analysis and its interpretation appropriate?

Not applicable

Are all the source data underlying the results available to ensure full reproducibility?

Yes

Is the study design appropriate and is the work technically sound?

Partly

Are the conclusions drawn adequately supported by the results?

Partly

Are sufficient details of methods and analysis provided to allow replication by others?

Yes

Reviewer Expertise:

arts, medical humanities, art history

I confirm that I have read this submission and believe that I have an appropriate level of expertise to confirm that it is of an acceptable scientific standard.

# Reviewer report: Reviewer response for version 3

**DOI:** 10.5256/f1000research.195407.r452892

**Published:** 2026-01-30

**Authors:** Margaret S. Chisolm

**Affiliations:** 1 Department of Psychiatry and Behavioral Services, and of Medicine, Johns Hopkins University School of Medicine, Baltimore, MD, USA

I appreciate the thoughtful response and revisions. No further comments

Is the work clearly and accurately presented and does it cite the current literature?

Partly

If applicable, is the statistical analysis and its interpretation appropriate?

Not applicable

Are all the source data underlying the results available to ensure full reproducibility?

Partly

Is the study design appropriate and is the work technically sound?

Partly

Are the conclusions drawn adequately supported by the results?

Partly

Are sufficient details of methods and analysis provided to allow replication by others?

No

Reviewer Expertise:

NA

I confirm that I have read this submission and believe that I have an appropriate level of expertise to confirm that it is of an acceptable scientific standard.

# Reviewer report: Reviewer response for version 2

**DOI:** 10.5256/f1000research.175471.r435754

**Published:** 2026-01-02

**Authors:** Sven Dupré

**Affiliations:** 1 Utrecht University, Utrecht, The Netherlands

This article convincingly shows that there is no sufficient proof that visual art observation is effective in novice healthcare professionals learning technical skills. Yet, it is less convincing in its choice of premises for the design of the study and how they relate to the representation of the literature. There is a rising interest in the role of the arts in medical education. Yet, the field is still in its infancy and in a explorative phase. The current article presents two demarcations, partly in response to comments of previous reviewers, which are of such a nature that it would be important to more clearly flag that they are not representative of the field or the literature in its entirety.

 First, in response to previous reviewers, the author has broadened the scope from diagnostic accuracy to technical skills. Yet, learning technical skills is only a part of the beneficial effects of art observation on medical education as listed in the literature. It is not possible to extrapolate conclusions on technical skills to the role of the arts in medical education as a whole, and it it important to explicitly state the limitations of the present study in the introduction and conclusion.

 Second, in response to previous reviewers, the focus is on novices, excluding experts or more experienced healthcare professionals. Yet, the literature only partly considers novices and the role of art observation in their acquiring of technical skills. In fact, it would be quite surprising that art observation would play a substantial role in developing diagnostic and technical skills among novices. As such, the conclusions reached in this article are not really surprising.

Is the work clearly and accurately presented and does it cite the current literature?

Partly

If applicable, is the statistical analysis and its interpretation appropriate?

Not applicable

Are all the source data underlying the results available to ensure full reproducibility?

Yes

Is the study design appropriate and is the work technically sound?

Partly

Are the conclusions drawn adequately supported by the results?

Partly

Are sufficient details of methods and analysis provided to allow replication by others?

Yes

Reviewer Expertise:

arts, medical humanities, art history

I confirm that I have read this submission and believe that I have an appropriate level of expertise to confirm that it is of an acceptable scientific standard, however I have significant reservations, as outlined above.

# Response

**Published:** 2026-01-17

**Authors:** Koji MATSUMOTO

**Affiliations:** Nagoya Gakuin University, Nagoya, Aichi Prefecture, Japan

Dear Reviewer,

 Thank you very much for your thorough review.

 As stated in the Introduction, this article focuses on the role of visual art observation in developing observation-related technical skills. Any remaining concerns you may have are beyond the scope of the article. I did not intend to extrapolate the conclusions to technical skills other than observation-related ones, nor to the role of the arts in health professional education. I also believe that no statement misleads readers into thinking that I made any such claims. Furthermore, as I discussed elsewhere (See Ref 1), I believe that the arts can play many roles in health professional education.

 However, as it may be beneficial for readers who are unfamiliar with this topic, I added a paragraph to the end of the Limitations section to address the points you raised:

 Finally, in health professional education for both novices (pre-service) and experts (in-service), the arts can play many roles beyond enhancing observational skills. ^115 – 122^ However, discussing such roles is outside the scope of this article. The author does not intend to extrapolate the conclusions to technical skills beyond those related to observation, nor the role of the arts in health professional education.

 [Ref 1] Matsumoto K. The use of arts in health professions education: applicability as a new teaching method. *Igaku Kyoiku / Medical Education (Japan)*. 2023;54(3):235-243. Article in Japanese.

# Reviewer report: Reviewer response for version 2

**DOI:** 10.5256/f1000research.175471.r371695

**Published:** 2025-03-26

**Authors:** Margaret S. Chisolm

**Affiliations:** 1 Department of Psychiatry and Behavioral Services, and of Medicine, Johns Hopkins University School of Medicine, Baltimore, MD, USA

Approved with no further suggestions

Is the work clearly and accurately presented and does it cite the current literature?

Partly

If applicable, is the statistical analysis and its interpretation appropriate?

Not applicable

Are all the source data underlying the results available to ensure full reproducibility?

Partly

Is the study design appropriate and is the work technically sound?

Partly

Are the conclusions drawn adequately supported by the results?

Partly

Are sufficient details of methods and analysis provided to allow replication by others?

No

Reviewer Expertise:

NA

I confirm that I have read this submission and believe that I have an appropriate level of expertise to confirm that it is of an acceptable scientific standard.

# Reviewer report: Reviewer response for version 1

**DOI:** 10.5256/f1000research.141887.r292047

**Published:** 2024-06-21

**Authors:** Margaret S. Chisolm

**Affiliations:** 1 Department of Psychiatry and Behavioral Services, and of Medicine, Johns Hopkins University School of Medicine, Baltimore, MD, USA

Abstract

 The abstract is lacking in conceptual clarity/relevance and presentation of replicable methods and clearly stated results. The aim of this article as stated is to review intervention studies that used "appreciation" of visual arts to foster observation skills AND discussed impact on diagnostic accuracy. This aim contains at least two concepts that are problematic for me: 1) the use of the word "appreciation" as most visual arts-based methods used in medical education are not concerned with art appreciation and 2) enhancement of observation skills  is just one of many targeted skills and attributes that may improve patient outcomes, via diagnostic accuracy or other mechanisms, all of which are more "downstream." Thus, I'm concerned with the basic premise as articulated of the manuscript. However, putting that aside for the moment, and turning to the methods sentence of the abstract. This is not a systematic review yet the abstract implies it is as it includes the term "previous systematic reviews." Systematic reviews need to follow PRISMA guidelines, which this review does not. It has no clearly stated inclusion/exclusion criteria, database names, search terms, duplicate review system, no flow chart, no clear search start and end dates, no exact numbers of articles reviewed etc. this lack prevents replication and limits assessment of strength of evidence and conclusions that can be drawn. Again, the aim of affects of "appreciating" art on "diagnosis" accuracy are questionable aims to begin with and lack of rigor in review methods makes this manuscript's conclusions more problematic. Moving to the results section of the abstract, few results are presented and this is more of a discussion section making it challenging to assess whether conclusions are supported by evidence. The conclusions section of the abstract is not supported by results as presented in abstract. No presentation of evidence for medical cases/knowledge to impact learning as compared to visual art "appreciation." No evidence presented in results section of abstract to support verbalization skills enhancement, etc. In conclusion, the abstract is lacking in coherence and clarity.

 Introduction 

 The manuscript would be enhanced by a clear articulation of the problem(s) that visual arts-based teaching is trying to solve and what the current non-visual arts-based methods are to address this problem. It would also be important to mention the international consensus that the arts and humanities are fundamental to medical education (as stated by WHO, NASEM and AAMC FRAHME reports) and the Moniz et al scoping review [Ref 1] and two papers on the Prism model describing the functions that the arts and humanities have served in medical education to date.

 Although one of the primary goals of developing observations skills is to improve diagnostic accuracy, this is a very downstream outcome to which many other skills may contribute and also other downstream outcomes are clinically relevant as well (e.g., being able to observe nonverbals and so enhance communication skills to build rapport and enhance adherence with treatment recommendations, as one example). Also it appears that results are included in introduction prior to presenting methods and results later, and would avoid including results in the introduction and instead put this in context of literature more broadly. A research question needs to be articulated.

 Methods

 The previously mentioned review studies should be placed in the results, not in the Introduction or Methods sections. The Methods section should include the search terms, the databases searched, the exact search date, the number of abstracts/titles identified in search, exact inclusion/exclusion etc as presented in PRISMA guidelines. The reader should know how many non-duplicate abstracts/titles were identified, how many of those were included in  full text review, etc.

 Results

 The word "around" should never be part of a systematic review, which this is not. More precision throughout is necessary. Not clear why the term "visual arts appreciation" is used as "appreciation" is not integral to teaching visual arts-based medical education. 

 The author mistates two of the three core questions of VTS. These are precisely researched and worded questions so getting two of them incorrect here is very concerning. These questions have the primary aim of holding the group in inquiry as well the aim as stated by the author of having the participants ground their inferences in visual evidence.

 The penultimate paragraph of the Results section was the most informative of the manuscript, describing the study design and outcomes, which are suggestive of impact on observation skills. More precision in the text would be helpful here (i.e., % studies versus "most" AAMC or "some"). 

 The last paragraph brings up diagnostic accuracy, which - again - the author declares as a primary aim of this work, but I'm not sure it is THE primary aim of any of the use of visual arts in medical education, and so it's not surprising that only one study measured this.

 Discussion

 Use of the word "target" in the first sentence to describe the work of art is an odd choice; perhaps just saying "artwork" would be clearer. The author raises some interesting ideas in this section. However the idea that observing for a longer period of time is not necessary for an accurate diagnosis among experts  obscures the important role of taking time for beginners (presumably the target learners for most of these exercises) to avoid premature diagnostic closure via arriving at a diagnosis too quickly and not staying open to possibilities is important to good patient outcomes. Plus even among experts who can quickly diagnose most presentations accurately, instances exist where taking more time would have resulted in a more accurate diagnosis for an individual patient. Similarly the ability to look should precede the ability to know what to discard as relevant, so a beginner's inability to overlook irrelevant features seems a bit irrelevant to teaching beginners to look. At least one more recent study does support visual arts to make a statistically significant contribution to tolerance for ambiguity (Tackett et al, Med Ed Online).[Rev 2]

 Again, the use of "appreciating" visual arts misses the mark of what visual arts-based teaching aims to do. Art appreciation is beside the point. 

 The author raises good questions about the limitations of transfer and the need for more research to study the transfer of skills from the museum or classroom to the clinic. They make a good point about the role of visual arts to foster communication skills (both listening and "verbalizing," which perhaps "speaking" or "articulately" would be a less awkward word choice) and team-building/perspective taking.

 Conclusions

 Although the methods/results presentation make it difficult to be sure that the conclusions reached are accurate, I would not be surprised by a lack of evidence on whether visual arts-based teaching aids diagnostic accuracy for the reasons outlined above. This is an emerging field whose focus has been on exploring the role of the arts in developing many clinically relevant skills and attitudes, which - taken as a whole - may support patient outcomes (including diagnosis and treatment planning) downstream. The field is in an exploratory phase and its aims are to explore an array of attributes that may have an effect on these and other "downstream"  outcomes.

 Limitations should include that this is not a systematic review in accordance with the PRISMA guidelines, and that relevant articles may have been missed by the search strategy etc

 Clearly more rigor is needed in the field of visual arts-based medical education research. The author does well to point this out and give some ideas for future directions. However, the findings of the study and these recommendations for improvement should be place in the context of the broader literature on these research gaps as outlined in the Howley et al AAMC FRAHME report and the Moniz et al scoping review, which also suggests more research rigor etc. I would disagree with several conclusions. The conclusion that "adopting visual art appreciation is not encouraged" seems to me an overstatement given the limitations of this review and the exploratory nature of the field. Clearly innovation is needed that goes beyond medical cases and knowledge transfer to teach students an array of otherwise difficult-to-teach clinically relevant attributes, which may improve patient outcomes, whether via

 diagnostic accuracy or other effects. I also see great value in increasing learners' awareness of what is an observation and what is an interpretation. The metacognitive skills of recognizing that an interpretation lacks visual evidence, raises awareness of possible biased thinking.

Is the work clearly and accurately presented and does it cite the current literature?

Partly

If applicable, is the statistical analysis and its interpretation appropriate?

Not applicable

Are all the source data underlying the results available to ensure full reproducibility?

Partly

Is the study design appropriate and is the work technically sound?

Partly

Are the conclusions drawn adequately supported by the results?

Partly

Are sufficient details of methods and analysis provided to allow replication by others?

No

Reviewer Expertise:

museum-based health professions education

I confirm that I have read this submission and believe that I have an appropriate level of expertise to confirm that it is of an acceptable scientific standard, however I have significant reservations, as outlined above.

## References

[1] : How Are the Arts and Humanities Used in Medical Education? Results of a Scoping Review. *Acad Med* .2021;96(8) : 10.1097/ACM.0000000000004118 1213-1222 10.1097/ACM.0000000000004118 33830951

[2] : Transformative experiences at art museums to support flourishing in medicine. *Med Educ Online* .2023;28(1) : 10.1080/10872981.2023.2202914 2202914 10.1080/10872981.2023.2202914 37074677 PMC10120547

# Response

**Published:** 2025-02-13

**Authors:** Koji MATSUMOTO

**Affiliations:** Nagoya Gakuin University, Nagoya, Aichi Prefecture, Japan

Dear Reviewer,

 Thank you very much for your thorough review. Your comments are really valuable and will contribute to improving the quality of the work.

 I designed a scoping reviews protocol in this revised version following the PRISMA Extension for Scoping Reviews (PRISMA-ScR). Inspired by your comments, I also expanded my focus from diagnostic skills to the technical skills that the intervention studies claimed art observation can foster.

 I would like to address each of the points you raised (some of the comments are reordered, and duplicate comments were consolidated):

**Comment (C):** The abstract is lacking in conceptual clarity/relevance and presentation of replicable methods and clearly stated results.

**Response (R): **I designed the study following the PRISMA-ScR and revised the entire Abstract.

**C:** Not clear why the term "visual arts appreciation" is used as "appreciation" is not integral to teaching visual arts-based medical education.

**R: **According to a suggestion by another reviewer, I modified it to “visual art observation.”

**C:** It would also be important to mention the international consensus that the arts and humanities are fundamental to medical education.

**R: **I did not discuss this in this paper but did elsewhere (See Ref 1). I believe I understand this background.

 [Ref 1] Matsumoto K. The use of arts in health professions education: applicability as a new teaching method. *Igaku Kyoiku / Medical Education (Japan)*. 2023;54(3):235-243. Article in Japanese.

**C:** Enhancement of observation skills is just one of many targeted skills and attributes that may improve patient outcomes, via diagnostic accuracy or other mechanisms, all of which are more "downstream."

**R: **I agree; this paper examines the claim that the interventions also work “downstream,” as the advocates argued.

**C:** Although one of the primary goals of developing observations skills is to improve diagnostic accuracy, this is a very downstream outcome to which many other skills may contribute and also other downstream outcomes are clinically relevant as well.

 Then, I added the “Technical knowledge and skills based on observation skills” subsection to insist that it is necessary to consider the technical knowledge and skills of the learners.

**C:** I'm not sure to improve diagnostic accuracy is THE primary aim of any of the use of visual arts in medical education, and so it's not surprising that only one study measured this.

**R: **The scope of the study extended to skills related to observation.

**C:** It appears that results are included in introduction prior to presenting methods and results later, and would avoid including results in the introduction and instead put this in context of literature more broadly.

**C:** The previously mentioned review studies should be placed in the results, not in the Introduction or Methods sections.

**R:** The reason for mentioning the prior reviews in the Introduction is to show the need for this review by presenting the attainments and unsolved issues of the previous reviews.

**C:** A research question needs to be articulated.

**R:** I added this to the Introduction.

**C:** The Methods should follow PRISMA guidelines.

**R: **I revised the Methods according to the PRISMA-ScR.

**C:** The word "around" should never be part of a systematic review, which this is not. More precision in the text would be helpful here (i.e., % studies versus "most" AAMC or "some").

**R:** I followed this suggestion and revised this section accordingly.

**C:** The author misstates two of the three core questions of VTS.

**R:** I revised this, thank you for pointing it out.

**C:** The penultimate paragraph of the Results section was the most informative of the manuscript, describing the study design and outcomes, which are suggestive of impact on observation skills.

**R:** In this revised version, the description was expanded in more detail.

**C:** In Discussion, use of the word "target" in the first sentence to describe the work of art is an odd choice.

**R:** In this version, this sentence was entirely revised.

**C:** The idea that observing for a longer period of time is not necessary for an accurate diagnosis among experts obscures the important role of taking time for beginners to avoid premature diagnostic closure via arriving at a diagnosis too quickly and not staying open to possibilities is important to good patient outcomes. Instances exist where taking more time would have resulted in a more accurate diagnosis for an individual patient.

**R:** I revised the subsection “Longer periods of careful and systematic observations for accurate diagnosis” to state clearly that teaching novice medical students systematic viewing is not helpful. The previous studies cited in the subsection deny the idea. Additionally, please tell me the specific references you are referring to.

**C:** At least one more recent study does support visual arts to make a statistically significant contribution to tolerance for ambiguity.

**R:** As mentioned in the “Longer periods of careful and systematic observations for accurate diagnosis” subsection, the effectiveness of tolerance to clinical practice is debatable.

**C:** The ability to look should precede the ability to know what to discard as relevant, so a beginner's inability to overlook irrelevant features seems a bit irrelevant to teaching beginners to look.

**R:** I understood this suggestion to mean that teaching students how to look and then the knowledge or skills to identify the critical signs may be beneficial. If this is what you mean, I discussed this as scaffolding in the “Long-term effects such as scaffolding” subsection, as I did in version 1.

**C:** The author raises good questions about the limitations of transfer and the need for more research to study the transfer of skills from the museum or classroom to the clinic.

**R:** This was mentioned in the revised version as well.

**C:** They make a good point about the role of visual arts to foster communication skills (both listening and and team-building/perspective taking.

**R: **As a result of reanalysis, I stated that there is insufficient evidence that art observation helps with these skills in the Results and “Fostering verbalizing skills…” subsection of the Discussion.

**C:** "verbalizing," which perhaps "speaking" or "articulately" would be a less awkward word choice.

**R:** “Vernalizing” means making words out of what one sees, as the text states. Additionally, given the use of description as an outcome measure in the studies, I think “speaking” and “articulately” differ slightly from what I wished to express.

**C:** I would not be surprised by a lack of evidence on whether visual arts-based teaching aids diagnostic accuracy. The field is in an exploratory phase and its aims are to explore an array of attributes that may have an effect on these and other "downstream" outcomes.

**C:** The conclusion that "adopting visual art appreciation is not encouraged" seems to me an overstatement given the limitations of this review and the exploratory nature of the field.

**C:** The findings of the study and these recommendations for improvement should be place in the context of the broader literature on these research gaps as outlined in the Howley et al AAMC FRAHME report and the Moniz et al scoping review, which also suggests more research rigor etc.

**C:** Clearly innovation is needed that goes beyond medical cases and knowledge transfer to teach students an array of otherwise difficult-to-teach clinically relevant attributes, which may improve patient outcomes, whether via diagnostic accuracy or other effects.

**R:** I agree with these opinions and revised the Discussion and Conclusion from this standpoint. I tried to suggest directions and challenges for future research for developing this field. On the other hand, I did not provide suggestions for practice, judging that there is not enough evidence to apply them to practice.

**C:** Limitations should include that this is not a systematic review in accordance with the PRISMA guidelines, and that relevant articles may have been missed by the search strategy etc

**R:** I designed the study according to the PRISMA-ScR.

# Reviewer report: Reviewer response for version 1

**DOI:** 10.5256/f1000research.141887.r239810

**Published:** 2024-02-16

**Authors:** Nicole J Fernandez

**Affiliations:** 1 University of Saskatchewan, Saskatoon, Saskatchewan, Canada

This paper is a welcome addition to the literature on the humanities in medical education and attempts the daunting task of determining if art observation has a positive impact on medical diagnosis in novice learners. The approach to this question is logical, although in some areas, the reader would benefit from more explanation and context. The conclusion that there is little evidence to support improved diagnosis as a result of art observation is well-supported, although the broad reasons for this could be further detailed, and the statement that another approach would be better is outside the scope of the article.

 The article could be improved by considering these further comments to the author:

 Title: Use of “appreciating” -consider re-wording. This implies a casual approach to looking at art, while slow looking is an iterative rigorous process. Perhaps “art observation” instead of “appreciating visual arts”. Also “medical diagnostic skills” instead of “medical diagnosis skills”. Suggest including ‘who’ as well- medical students? Novice learners?

 Possible revised title: Art observation may not improve medical diagnostic skills in novice learners

 The writing style is inconsistent and unusual in some places. Usually in English scientific writing “I” or “the author” is not used- recommend rephrasing throughout. (For example: “The author collected information on…” becomes “Information was collected on…”.)

 Abstract: The Background section of an abstract usually provides context for the study rather than describing what the study did.

 Make sure that any changes to the body of the article are reflected in the abstract (eg changes to the conclusions).

 There are opportunities throughout to provide more context and help the reader’s understanding of the subject- some of these are noted below. Being explicit is important- there may be assumptions that are not shared by all readers, especially regarding definitions and terms used.

 -Define what you mean by intervention/empirical intervention study. This is important in helping the reader to know which studies you considered.

 -Clarify what you mean by diagnosis- visual diagnosis only (eg radiology, gross pathology)? Clinical reasoning (which would be much broader)?

 -Specifically outline your inclusion and exclusion criteria

 -How did you decide about diagnostic evidence? Is improved diagnostic accuracy the only criterion? What about other elements of the diagnostic process or approach to complex cases in which the diagnosis is unclear?

 -The term ‘expertization’ may not be familiar to readers and should be explicitly defined, including what is meant by ‘expertization studies’.

 -In Methods, “features and tendencies” of the selected articles is mentioned- please explain what is meant by this.

 The final paragraph of the introduction is not needed, as this is the standard format for a journal article.

 Methods-

 The first paragraph mentions “studies that used visual arts for cultivating observation skills” but was this the only goal of the included studies? This does not seem consistent with what is stated elsewhere in the article.

 3 ^rd^ paragraph, second sentence “were excluded” needs to be moved to outside the parentheses

 Suggest including a figure that shows how articles were selected, numbers of articles at each step of the process, that 12 articles were selected for full review. How many articles were found using the review articles vs by manual search? There are many examples of figures like this in the published literature.

 Results-

 Abbreviations need to be explained in the table (regardless of whether they are also explained in the text- the table should be able to stand alone).

 Results could be strengthened by using numbers instead of terms like “most”

 Discussion-

 -Overall the discussion would benefit from a clearer focus on the original question- specifically diagnostic skills, not art observation studies in general.

 -Why might improvement in diagnostic skills be difficult to demonstrate in a study? Provide context, difficulty of assessing long term effects. How could future researchers attempt this? (currently in conclusions)

 -Consider that one might not see an improvement right after the intervention in 2 ^nd^ year, but could it be helpful once students have more diagnostic experience, eg in 4 ^th^ year?

 -Clearly and briefly list what reported benefits of art observation are- the information is there, but scattered. It could be condensed, as this is not the original focus of the paper.

 -If included studies were only those designed to improve observation skills, is it reasonable to assess them for improvement in diagnostic skills? How strong can any conclusions be? Only 1 study assessed diagnostic accuracy, and this was in a limited domain.

 The relevance of expertization studies is unclear, since the art observation interventions were done with novice learners. Novices are not experts, one can’t teach them the same way. Just because experts are more likely to misdiagnose if they take longer, it doesn’t necessarily follow that the same is true for novices. If your position is that novices should be taught like experts, this needs to be explicitly stated and supported from the literature.

 Throughout the discussion it needs to be made clear when you are referring to studies in experts vs studies in novices. This is not clear for a reader who is not already familiar with this literature.

 The Discussion covers much material that does not seem closely related to the original goal and could be condensed and refined to focus on diagnostic skills. It is important to emphasize that only 1 art observation study explicitly looked at diagnosis, and did this in a limited way. The conclusion appears to be that the impact of art observation on diagnostic skill has not been adequately investigated, rather than that it is ineffective. I don’t agree that it has been “shown that appreciating visual arts may not have a direct effect on making an accurate diagnosis”; the statement “there is no concrete evidence on whether appreciating visual art contributes toward an accurate diagnosis” is more accurate.

 Conclusions should be a single paragraph stating your major findings- the section titled Conclusions is not really a conclusion. This material is more appropriate in the Discussion.

 The claim that “it would be better to…” was not the focus of this article- a review of this literature would need to be included in order to support this. It could be suggested that if improving diagnostic accuracy is the single goal, there may be other methods that are more effective than art observation. But is this the only goal of art observation interventions? I don’t believe so. Observation is the first part of the diagnostic reasoning pathway, but not the only part, certainly not a panacea. It seems simplistic to expect a single intervention to have a great and immediate impact on diagnostic accuracy.

 Limitations:

 -No veterinary medicine literature included, although nursing literature was included. Is there a reason for this? There are several relevant articles in the vet med literature.

 -Studies that included other methods were excluded- can’t comment on what the effects of these studies are

 -In methods you mention the limitations of eg thematic analysis, but there is no mention of the limitations of using quantitative population statistics

Is the work clearly and accurately presented and does it cite the current literature?

Partly

If applicable, is the statistical analysis and its interpretation appropriate?

Not applicable

Are all the source data underlying the results available to ensure full reproducibility?

Yes

Is the study design appropriate and is the work technically sound?

Partly

Are the conclusions drawn adequately supported by the results?

Partly

Are sufficient details of methods and analysis provided to allow replication by others?

Partly

Reviewer Expertise:

Tolerance of ambiguity, teaching observational skills, case-based learning, clinical pathology

I confirm that I have read this submission and believe that I have an appropriate level of expertise to confirm that it is of an acceptable scientific standard, however I have significant reservations, as outlined above.

# Response

**Published:** 2025-02-13

**Authors:** Koji MATSUMOTO

**Affiliations:** Nagoya Gakuin University, Nagoya, Aichi Prefecture, Japan

Dear Reviewer,

 Thank you very much for your thorough review. Your comments are really valuable and will contribute to improving the quality of the article.

 I added the scoping reviews protocol in this revised version following the PRISMA Extension for Scoping Reviews (PRISMA-ScR). I expanded my focus from diagnostic skills to the technical skills that the intervention studies claimed art observation can foster. Therefore, I cannot sufficiently respond to your suggestions regarding diagnostic skills. However, inspired by the suggestions, I discussed the relationship between visual art observation and technical knowledge and skills acquired through education and training.

 I would like to address each of the points you raised (some of the comments are reordered, and duplicate comments were consolidated):

**Comment (C):** In the title, use “art observation” instead of “appreciating visual arts”. Also “medical diagnostic skills” instead of “medical diagnosis skills”. Suggest including ‘who’ as well- medical students? Novice learners?

**Response (R):** I modified the title: “Effects of visual art observation on technical skills in novice healthcare learners: A scoping review.” I also revised the language to use “visual art observation” and “diagnostic skills” in the article. Moreover, I added the definition of novice medical learners in 4 ^th^ paragraph of the Introduction: “novice healthcare learners, namely, students, residents, trainees, or inexperienced workers in related majors or domains.”

**C:** Usually in English scientific writing “I” or “the author” is not used- recommend rephrasing throughout.

**R:** I appreciate this feedback. In some cases, I used the personal pronoun or the subject “the author” to avoid passive sentence construction. I tried to use the passive form as little as possible, according to the guidelines of some journals on medical education.

**C:** In the Abstract, The Background section of an abstract usually provides context for the study rather than describing what the study did.

**R:** I modified this, as you pointed out.

**C:** Make sure that any changes to the body of the article are reflected in the abstract.

**R: **I also modified the abstract.

**C:** Define what you mean by intervention/empirical intervention study.

**R:** I added the definition in 1st paragraph of the Introduction: “studies which include both training practices and corroborating its effects.”

**C:** “Expertization” should be explicitly defined.

**R: **I deleted the word and removed it from the article.

**C:** In Methods, “features and tendencies” of the selected articles is mentioned- please explain what is meant by this.

**R: **I revised research question one to make it clearer and more concise: What key features or trends characterize visual art observation training in intervention studies aimed at improving observation skills?

**C:** In Methods, the first paragraph mentions “studies that used visual arts for cultivating observation skills” but was this the only goal of the included studies? This does not seem consistent with what is stated elsewhere in the article.

**R: **As stated in Methods, I included the studies that primarily aimed to “evaluate the effect of the intervention on technical skills related to observation,” and the goals that the included studies stated clearly were listed in Table 2.

**C:** The final paragraph of the introduction is not needed.

**R: **The paragraph was written at the journal editor’s suggestion.

**C:** In Methods, 3rd paragraph, second sentence “were excluded” needs to be moved to outside the parentheses

**R: **These words were removed.

**C: **Specifically outline your inclusion and exclusion criteria. Include a figure that shows how articles were selected, numbers of articles at each step of the process, that 12 articles were selected for full review.

**R: **Following the PRISMA-ScR, I added information to the Methods and added Figure 1.

**C: **Abbreviations need to be explained in the table (regardless of whether they are also explained in the text- the table should be able to stand alone).

**R: **I modified Table 2 as per your recommendation.

**C: **Results could be strengthened by using numbers instead of terms like “most”

**R: **I modified this, as you pointed out.

**C: **Overall the discussion would benefit from a clearer focus on the original question- specifically diagnostic skills, not art observation studies in general.

**R: **As explained, the focus has changed.

**C: **Clarify what you mean by diagnosis- visual diagnosis only? Clinical reasoning (which would be much broader)? How did you decide about diagnostic evidence? Is improved diagnostic accuracy the only criterion? What about other elements of the diagnostic process or approach to complex cases in which the diagnosis is unclear?

**R: **Due to the change in the focus of this paper, I cannot directly answer these questions. On the other hand, as mentioned in the Introduction, the intervention studies claimed that the observational skills fostered by art observation could be applied to both medical imaging and clinical interviews. See also the next response.

**C: **If included studies were only those designed to improve observation skills, is it reasonable to assess them for improvement in diagnostic skills? How strong can any conclusions be? Only 1 study assessed diagnostic accuracy, and this was in a limited domain.

**C: **Observation is the first part of the diagnostic reasoning pathway, but not the only part. It seems simplistic to expect a single intervention to have a great and immediate impact on diagnostic accuracy.

**R: **I agree with you. However, the intervention studies claimed that art observation could facilitate diagnostic skills (directly or indirectly), as mentioned in the Introduction. This paper examined the claim. Moreover, in the “Technical knowledge and skills based on observation skills” subsection of the Discussion section, I argued that the relationship to technical knowledge and skills of learners should be considered to examine this clearly.

**C: **Why might improvement in diagnostic skills be difficult to demonstrate in a study? Provide context, difficulty of assessing long term effects. How could future researchers attempt this?

**R: **See the response immediately above. In addition, I added the subsection “Long-term effects such as scaffolding” to discuss this. See also the “Implications for future research” subsection.

**C: **Throughout the discussion it needs to be made clear when you are referring to studies in experts vs studies in novices. This is not clear for a reader who is not already familiar with this literature.

**R: **This seems to relate to the next comment. If not, I may have misunderstood your question. Please let me know if the subsequent response is not satisfactory, and I will do my best to address your question.

**C: **Novices are not experts, one can’t teach them the same way. If your position is that novices should be taught like experts, this needs to be explicitly stated and supported from the literature.

**R: **The revised version does not mention that novices should be taught like experts. Concerning this comment, I also revised the subsection “Longer periods of careful and systematic observations for accurate diagnosis” to state clearly that teaching novice medical students systematic viewing is not helpful.

**C: **Consider that one might not see an improvement right after the intervention in 2nd year, but could it be helpful once students have more diagnostic experience, eg in 4th year?

**R: **This is discussed in the subsection “Long-term effects such as scaffolding.”

**C: **Clearly and briefly list what reported benefits of art observation are.

**R: **In the 2 ^nd^ paragraph of the Introduction, I listed the technical skills that intervention studies aimed at. I also added this as the result of this review in the subsection “Outcome measures and their results” in the Results section.

**C: **Conclusions should be a single paragraph stating your major findings.

**R: **I modified it as you pointed out.

**C: **The conclusion appears to be that the impact of art observation on diagnostic skill has not been adequately investigated, rather than that it is ineffective. The statement “there is no concrete evidence on whether appreciating visual art contributes toward an accurate diagnosis” is more accurate.

**R: **As a result of the reanalysis, such wording was used in the paper.

**C: **In Conclusions, the claim that “it would be better to use learning modules based on perceptual learning using cases to foster pattern recognition” was not the focus of this article.

**R: **This description was deleted.

**C: **No veterinary medicine literature was included. Is there a reason for this?

**R: **Three studies in veterinary education were included because they were comparable to training and experimental designs in human health professional education.

**C:** In Limitations, studies that included other methods were excluded- can’t comment on what the effects of these studies are. Also, there is no mention of the limitations of using quantitative population statistics.

**R: **I added these to the Limitations subsection.
